# Ground improvement using chemical methods: A review

**DOI:** 10.1016/j.heliyon.2021.e07678

**Published:** 2021-07-28

**Authors:** Harshal Verma, Arunava Ray, Rajesh Rai, Tushar Gupta, Neeraj Mehta

**Affiliations:** a Department of Mining Engineering, IIT(BHU), Varanasi, Uttar Pradesh 221005, India; b Department of Mining Engineering, National Institute of Technology Rourkela, Odisha 769001, India; c Department of Physics, Institute of Science, Banaras Hindu University, Varanasi, Uttar Pradesh 221005, India

**Keywords:** Ground improvement, Chemical stabilisers, Biochemical stabilisers, Electrochemical stabilisers, Inorganic and organic stabilisers

## Abstract

Ground improvement will be critically important in the present and future geotechnical practice for designing the structures in weak soil. This paper presents a review of the recent development in ground improvement techniques, especially chemical stabilisers. Various available chemical stabilisers are identified and compared with other available methods. Though the use of chemicals provides an excellent alternative to the traditional methods, they still lack proper understanding regarding their use, handling, application, and long-term effect on the environment. Various chemical stabilisers and their applicability conditions are summarised in the present paper. Insight of biochemical, electrochemical, inorganic, and organic stabilisers is presented with future scope of these methods along with the potential areas where a lot of efforts is needed to industrialise these methods are also discussed briefly. A need for developing a more environmentally friendly and safe method was felt while reviewing these methods. Lack of a large amount of data is a major concern for lesser use of these methods industrially. A lot of laboratory and field experiments should be conducted in different conditions to ensure safe results from chemical stabilisers.

## 1 Introduction

Ground improvement has always been one of the major thrust areas of geotechnical engineering. It is vertically crucial in the design of the structure in weak soil. Before any development or construction work for either civil structures or mining activities, it is crucial to know the local soil type, present and future use of the land area, required strengths for holding the above structural loads, and estimated cost of the project (OnyeloweKen and Okafor, 2006). In case the soil of the selected site does not have desired structural properties, e.g., appropriate cohesion, internal angle of friction, bearing capacity, swelling factor, etc., it becomes necessary to improve these properties using external means. The effect of soil instability can be diverse, including cases of liquefaction, heaving, swelling, and plastic deformation (Dawson et al., 1998). The effects of unstable soil are correspondingly catastrophic, ranging from slope failures and foundation sinkage to total collapse of the tunnels and mine dumps, overlying buildings, and other structures (Gupta et al., 2019).

Several methods are used for improving the ground conditions for safe and reliable construction. Based on the treatment method, the engineering techniques of ground improvement can be broadly grouped into three categories: mechanical, biological, and chemical stabilisation. Among these, mechanical stabilisation is the most common and oldest technique of ground improvement where the soil's density is increased by the application of mechanical force and compacting the surface layers by static and dynamic loading (Patel, 2019; Guetif et al., 2007). Soil biological stabilisation techniques are the combined applications of engineering practices and ecological principles for designing and building a system that will contain living plant materials as the structural component. The biological method of stabilisation not only beneficial from the engineering point of view but is also beneficial because of the ecological and environment-friendly nature (Sotir, 2001; Wu et al., 1979). The intention is not to have an immediate effect; instead, develop a system that will be sustainable and will ensure long-term remediation (Vannoppen et al., 2015). In chemical stabilisation, ground improvement is achieved by mixing various chemicals with soil to develop desirable characteristics. Uses of Inorganic pozzolanic/cementitious binders like fly ash, cement, lime, or some calcium-based chemicals are some of the most commonly used chemical methods. These methods have shown a long-term change in ground properties, but usually, some degree of environmental concerns is associated with them (Gaafer et al., 2015). In modern times, various types of complex chemical polymers are being used to improve the soil. These polymers react to form strong polymeric structure binding soil particles and fill up the soil voids to strengthen the overall structure. Some of the popular polymeric chemical stabilisers include polyurethane, polyacrylamides, and poly-acrylates.

The ground improvement as a sub-branch of the Geotechnical Engineering domain has made considerable advances since the practices began to develop in the mid-20th century. Most techniques have undergone drastic changes in terms of application and optimisation. The present paper discusses the use of chemical stabilisers as ground improvement techniques which includes biochemical and electrochemical methods along with various admixture and chemical reagents. Several detail literature studies on the use of chemical stabilisers are present; however, all of them were limited to a particular specific soil type or a particular type of chemical stabiliser. For example, Zahri and Zainorabidin (2019) worked on soft soil using various traditional and non-traditional chemical stabiliser, including polymer and lignosulfonate. Similarly, Kazemian et al. (2011) and Kazemain and Barghchi (2012) worked on soft soil using various chemical stabiliser, including Sodium Silicate grout, cement stabilisation, fibre reinforcement and acrylamide, polyurethane, epoxy resins, lignosulphates respectively. Puppala and Pedarla (2017) studied lime, cement and biopolymer on the stabilisation of expansive soils. Xu et al. (2018) studied the effect of non-traditional chemical stabilisers including acids, enzymes, lignosulfonate, polymers, tree resins, petroleum emulsions, and salts on bauxite residue (red sand) dust control. Wang et al. (2017) reviewed microbial induced carbonate precipitation to stabilise ground in place of mechanical compaction and chemical grouting which possess several disadvantages including high cost, high energy consumption, and potential environmental pollution. Van Paassen (2011) gave an overview of the latest research and developments on bio-mediated ground improvement in the Netherlands, including the first pilot application of biogrouting to stabilise horizontal boreholes through gravel layers. Wani and Mir (2020) performed a comprehensive review on Microbial geo-technology in ground improvement techniques which included the study of different factors that affect the process of biological improvement overall including the type of microbes, the quantity of microbes used, cementation solution molarity, pH of the system, treatment method, temperature, degree of saturation, the density of soil, nutrient availability, etc. Similarly, Ashraf et al. (2017) gave a detailed review of soil improvement using microbial induced Carbonate precipitation and biopolymers, including the advantages and limitations in both these mineralisation methods. Putra et al. (2020) and Kavazanjian and Hamdan (2015) reviewed the enzyme-induced calcite precipitation as a ground-improvement technique for improving the shear strength of sandy soil. Wong (2004) presented two case studies involving Chemical Lime Piles and Dynamic Replacement in soft ground. Kumari and Xiang (2019) reviewed biologically based grout material obtained from Biomineralisation to prevent soil liquefaction for ground improvement and compared it with the conventional soil improvement practices.

The use of various chemical stabilisers comes with their own sets of advantages, disadvantages, and applicability conditions. A closer look into all of the past works reveals the absence of substantial and qualitative work on ground improvement covering all the present-day available chemical stabilisers under a single study. This paper tried to present a comparative analysis of different chemical stabilisers, their applicability and the associated limitations. The aim is to present a brief but broad view of different chemical stabilisers under a single umbrella. Major findings from the literature have been discussed in detail, along with the identification of critical research gaps and future scope of work. It also discusses the effect of stabilisers on different soils qualitatively and highlights the cases where these methods may fail to incorporate the desired output.

## 2 Ground improvement using chemical stabilisers

**Figure 1:**
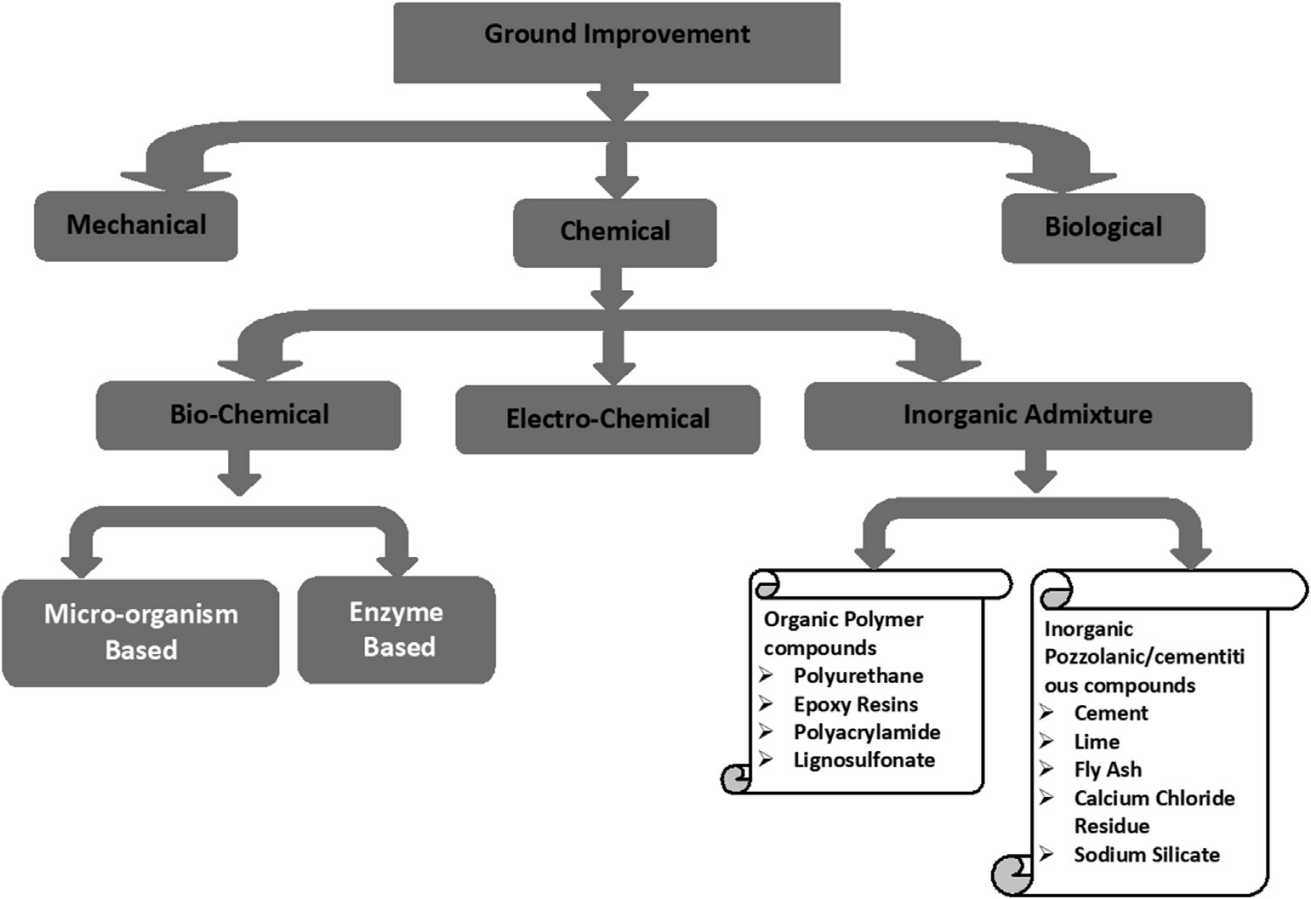
A flow-chart showing the chemical methods of ground improvement.

Modifications of the soil properties using physicochemical reactions prove to be more effective in sustaining the improvements over the long term than the other methods (Indraratna et al., 2015). Chemical processes such as mixing with cement, fly ash, lime, lime byproducts, chemical reagents, and blends of any one of these materials can be used to alter soil properties such as strength, compressibility, hydraulic conductivity, swelling potential, and volume change properties (Puppala et al., 1997; Puppala and Musenda, 2000). Foreign material can be added in-situ or ex-situ depending on the type of additive, design of the project, and equipment availability (Makusa, 2013). The chemicals generally used for stabilisation include industrial by-products or waste materials with cementation property. Several chemical additives have been developed in the last couple of decades; however, their selection and application are not uniform rather depend on the type of soil and other application factors. An individual additive acts differently with a different type of soil. Thus, detailed knowledge about the chemicals and their application requirements are of utmost importance. Based on nature and chemical composition, the chemical stabilisation methods are broadly divided into four major subgroups, as shown in Figure 1:

1. Biochemical methods
2. Electrochemical methods
3. Inorganic pozzolanic/cementitious material
4. Organic polymeric binders

### 2.1 Ground improvement using biochemical methods

Ground improvement using biochemical methods are divided into two major categories, Micro-biologically induced calcite precipitation (MICP) and Bio-enzymes.

#### 2.1.1 Microbiologically induced calcite precipitation (MICP)

**Figure 2:**
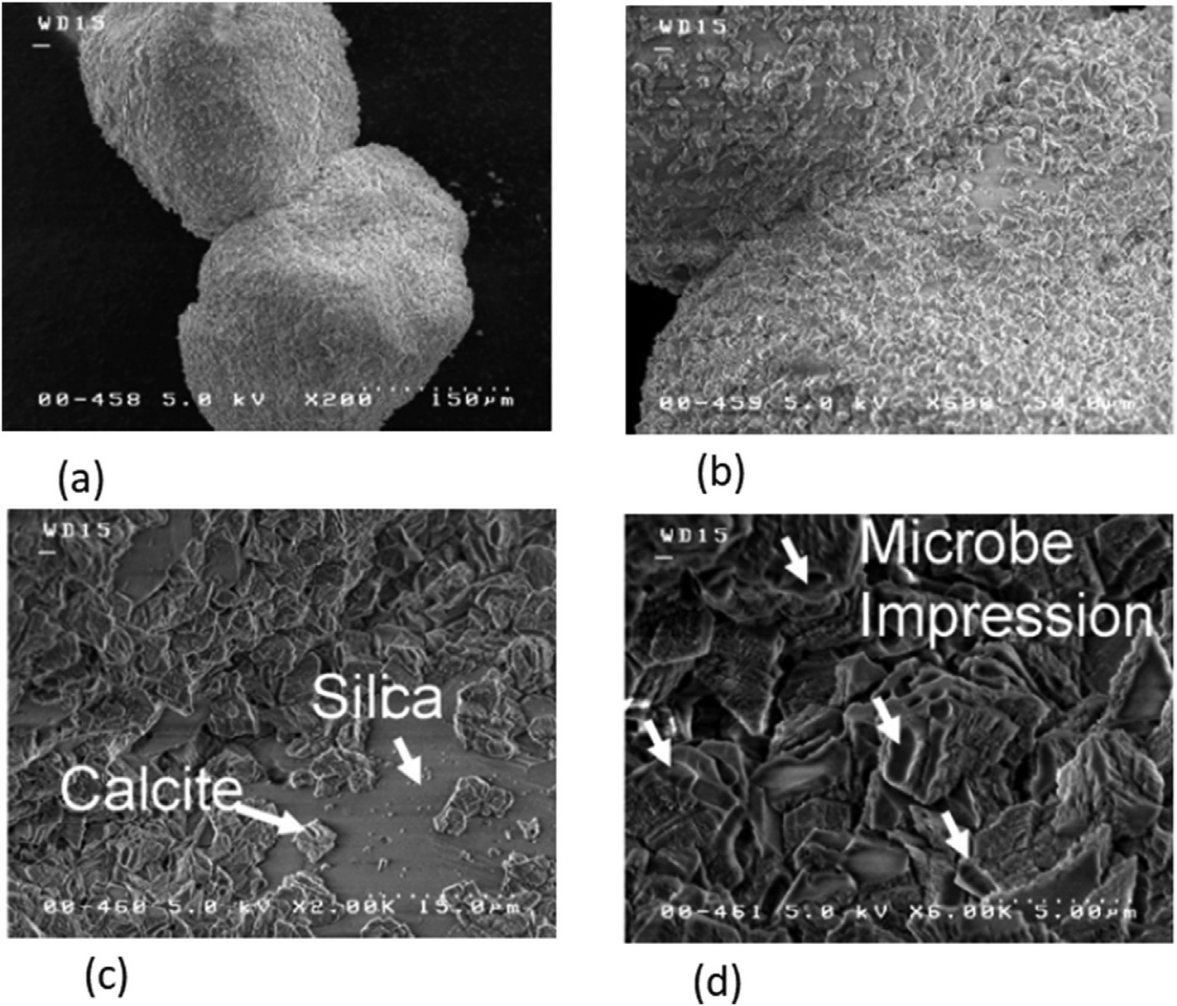
Images (a)–(d) shows the SEM images of the calcite precipitation at particle-particle contact and microbial habitat. Images (b) and (c) reveals that the calcite aggregates form a relatively heterogeneous structure that is feeble as compared to the silica particles (DeJong et al., 2010). Reprinted with permission from Elsevier.

The method is based on the precipitation of Calcium Carbonate (CaCO_3_) by the microorganism in an environmentally friendly way. Bacteria can survive over a wide range of environmental conditions, thus allowing their use as stabilisers efficiently. Bio-mineralization occurs at the site of nucleation by bacterial cell or by precipitation of CaCO_3_ due to an increase in alkalinity by ureolysis (Bibi et al., 2018). It can help in improving the ground condition either by bio-clogging or bio-cementation. Bio-clogging is filling of the pore space by CaCO_3,_ while bio-cementation is enhancing the strength of soil by increasing bond strength of soil particles by preferential calcite precipitation at particle-particle contact, as shown in Figure 2 (DeJong et al., 2010). Several microorganisms can precipitate CaCO_3_, but commonly ureolytic bacteria with some chemical reagent (to provide Calcium, e.g., CaCl_2_ and urea) are used. Urea is hydrolysed in the presence of urease enzyme, producing Ammonia and Carbon dioxide, while the medium's alkalinity rises during the reactions:

$${\text{CO}\left({\text{NH}}_{2}\right)}_{2}\left(\text{s}\right)+{\text{H}}_{2}\text{O }\left(\text{l}\right)\to 2{\text{NH}}_{3}\left(\text{aq}\right)+{\text{CO}}_{2}\left(\text{g}\right)\left[\text{rise in pH}\right]$$

$$2{\text{NH}}_{3}\left(\text{aq}\right)+{\text{CO}}_{2}\left(\text{g}\right)+{\text{H}}_{2}\text{O }\left(\text{l}\right)\to 2\text{N}{{\text{H}}_{4}}^{+}\left(\text{aq}\right)+\text{C}{{\text{O}}_{3}}^{2-}\left(\text{aq}\right)$$

The size of pore space in soil is also an important and limiting condition. Pore space between the particles of soil should be sufficient enough for allowing free movement of microbes (Achal et al., 2009). Thus, these methods are suitable for coarse-grain soil/sands and cementation of the sand column. The precipitation rate should not be too high to prevent blockage near the inlet and should not be too slow; otherwise, it will take a longer time to stabilise (Van Paassen et al., 2010). Cheng and Cord-Ruwisch (2014) identified clogging near the injection point resulting in treatment depth less than 1m for fine sand. Such a problem was not evident in the case of coarse sand. Moravej et al. (2018), while working with clays, deduced that precipitating agent (CaCl_2_) helps in reducing the dispersivity of soil by reducing the double layer thickness and thus decreases the erodibility of the soil.

A variety of microorganisms, including *Sporosarcina pasteurii, Idiomarina insulisalsae, Proteus vulgaris, Proteus mirabilis, Helicobacter pylori,* and Ureplasmas (Mocllicutes), can be used for the treatment of soil (Umar et al., 2016). DeJong et al. (2010) and Carmona et al. (2016) suggested laboratory mixing of urease with urea and Calcium Chloride or calcite precipitating chemicals which can be directly injected into the soil to escape through the hectic process of handling microbes. They found a reduction in the void ratio due to the precipitation of CaCO_3_. Usually, microbes prefer to stay near particle contact due to the availability of nutrients, and most of the calcite produced is near these zones, which is favourable for soil improvement. Cheng et al. (2014) suggested using saline water for Ca^2+^ ion precipitation instead of using chemicals. Although the rate of calcification was slow, however, it can significantly reduce the cost of the project.

Urease activity is a major factor determining the size of the crystal formed, which governs soil strength (Cheng et al., 2013). Cheng et al. (2017) studied various parameters on soil treated with *B. pasteurii* and found low urease activity resulting in the formation of larger aggregates that fill up the voids, thus result in higher unconfined compressive strength (UCS). They studied the addition of microbes over a wide range of temperatures (4 °C–50 °C); the optimum strength of ground was obtained at 25 °C. They also highlighted the problem arising from washing away immobile bacteria during and after precipitation, resulting in a decrease in the process's efficiency. Soon et al. (2013) used *B. megaterium* along with urea and Calcium Chloride (also containing nutrient broth, Ammonium Chloride, Sodium Bicarbonate in small quantity) as cementation reagent and identified an increase in shear strength and decrease in hydraulic conductivity of residual and sandy soil. Min Le (2015) showed the utility of MICP in the case of residual tropical soil, with more than 50% reduction in permeability and about 150% increase in shear strength. Lim et al. (2020) used fungi *Rhizopus oligosporus* with rice flour and reported an increase in soil cohesion due to the binding of particles by mycelium. They also found that 5% moisture content is the optimum moisture content for the growth of fungus since both dry and saturated conditions are not favourable for fungus growth.

Acid rain can severely decrease the effectiveness of biochemical stabilisation. Cheng et al. (2013) drew attention towards the vulnerability of bio-chemical stabilised soil towards acid rain and deduced around a 40% decrease in UCS of the top layer of the sand column due to acid rain. It was also observed that the decrease in permeability of bio-chemically treated soils was much lesser as compared to cement grouting. Permeability can affect the strength of the foundation by affecting the groundwater flow. An increase in pore water pressure due to reduced permeability could reduce the soil strength. Thus, one should plan their project by considering both acid rain and the hydrology of the area. Meyer et al. (2011) identified biochemical stabilisation as an environmentally friendly technique with the benefit of dust suppression.

S-wave velocity and Spectral Induced Polarization (SIP) test suggested by Saneiyan et al. (2018) to monitor the effectiveness and extent of stabilisation since it is difficult to predict the ultimate strength of bio-chemical stabilised ground and destructive methods are not practical. Wave velocity gives an insight into physical strength, while SIP can effectively identify activity due to its sensitivity towards the microbial cell. Changes in geophysical properties could also serve as an indirect measure of microbial processes and activities subsurface.

#### 2.1.2 Bio-enzyme

The use of Bio-enzyme also provides an alternative and economical method for soil stabilisation. Several Bio-enzymes are commercially available nowadays, e.g., Renolith, PermaZyme, TerraZyme, Fujibeton, etc. Their application is highly specific to the soil, e.g., Renolith is used to increase the tensile strength of granular soil, PermaZyme increase cohesion of silt and clay, Fujibeton increase UCS and California bearing ratio (CBR) of soil, and TerraZyme enhance the load-bearing capacity of fine-grain soil (Rajoria and Kaur, 2014). TerraZyme is one of the common enzymes extracted from vegetables and has no adverse effect on the environment. Navale et al. (2019) concluded that TerraZyme forms a cementitious material by reacting with organic matter present in the soil, which is responsible for the reduction in permeability and reduces swelling and improves the strength of the soil. Gupta et al. (2017), while working with clayey soil, deduced that TerraZyme could neutralise electrostatic charge around clayey particles. Thus, the soil could be compacted at a lower effort. Agarwal and Kaur (2014) used TerraZyme with black cotton soil and concluded a reduction in the swelling and permeability of soil with substantially increasing the compaction and UCS of soil.

Velasquez et al. (2006) highlighted the relationship between soil and the purpose-specific applicability of enzymes, primarily based on the clay and fine content. A particular type of enzyme could be beneficial for a particular soil type while it could be ineffective for other soils. Khan et al. (2015) used EarthZyme and TerraZyme enzymes on illite soil but did not find any significant increase in strength. However, some samples showed an increase in UCS, but that was due to increased density. Jamal and Kumar (2016) utilised Renolith with cement for stabilising the expansive black cotton soil and noticed a significant decrease in the liquid limit. Ganapathy et al. (2017) performed experiments with clayey sand and analogue it with the strong termite houses formed with the enzyme's help. Enzymes in the soil reduce double layer thickness by nullifying the charge of hydrogen ion of absorbed water molecules, reducing the soil's plasticity. They found a decrease in density and permeability and an increase in strength. The decrease in hydraulic conductivity was attributed to a reduction in void space in the soil due to the occurrence of microbial clusters.

Biochemical methods are adopted mainly due to environmental friendliness, cost-effective economics, and work without producing any noise (Cheng et al., 2017). Apart from subsurface stabilisation, the excreted extracellular polymeric substance by the bacteria at the soil surface helps bind the surface soil, reducing the vulnerability towards erosion. When this film is formed in the subsurface, it can significantly reduce or regulate the permeability of soil (DeJong et al., 2014). Also, some bacteria generate biogas, some of which gets entrapped into the voids, thus reducing the swelling and water holding capacity of the soil, the compressibility of soil increases which could be helpful in case of dynamic loading and liquefaction of sand.

### 2.2 Ground improvement using the electrochemical method

Electro-osmosis or electrochemical methods are beneficial for the consolidation and dewatering of fine-grain low permeability soils. However, its use as an effective ground improvement technique is in a nascent stage. When an electric potential difference is applied across two electrodes placed in soil, water moves toward the cathode. This method can be utilised to fill voids with colloidal chemicals or gels (Pamukcu and Winterkorn, 1991). The voids present in soil contain water with mobile positively charged ions. On the application of the electric field, ions flow and also drag water molecules together toward the cathode. For the selection of electrodes, copper provides the best option since graphite is readily oxidised by liberated heat and oxygen, steel and aluminium corrode, and conductivity decrease, and other metals such as silver are expensive. In the case of Copper, Copper Oxide and Hydroxide are formed, which are good conductors of electricity and prevent corrosion (Lo et al., 1991).

Electrical vertical drains (EVD) are used to overcome electric loss due to gas accumulation and corrosion at the electrode-soil interface. These are conducting polymer covering the metal (usually copper) electrodes (Karunaratne, 2011). The development of EVDs has significantly reduced the cost of operation (Martin et al., 2019). Estabragh et al. (2014) conducted consolidation tests over low plasticity clay using EVD and found 11- and 13-mm settlements from 15 and 45V, respectively. The results were far better than a 1mm settlement which was observed in the case of preloading with 22kPa. The settlement was more in the case of 45V, but the process's efficiency decreases with an increase in voltage. Mitchell (1993) quantified the effectiveness of the method in a term as a coefficient of electro-osmotic permeability, k_e_ as:

(1) $${\text{Q}}_{\text{e}}={\text{k}}_{\text{e}}{\text{i}}_{\text{e}}\text{A}$$

Where Q_e_ is the rate of water flow, A is the cross-section area, and, i_e_ is the voltage gradient. k_e_ of order 10^−9^m^2^/sV is found useful for stabilisation of clay. The following formular for determination of negative pore water pressure (u_e_) developed during the process was proposed by (Kaniraj et al., 2011):

(2) $${\text{u}}_{\text{e}}=\frac{-{\text{k}}_{\text{e}}{\text{Υ}}_{\text{w}}{\text{i}}_{\text{e}}\text{x}}{{\text{k}}_{\text{h}}}$$

**Figure 3:**
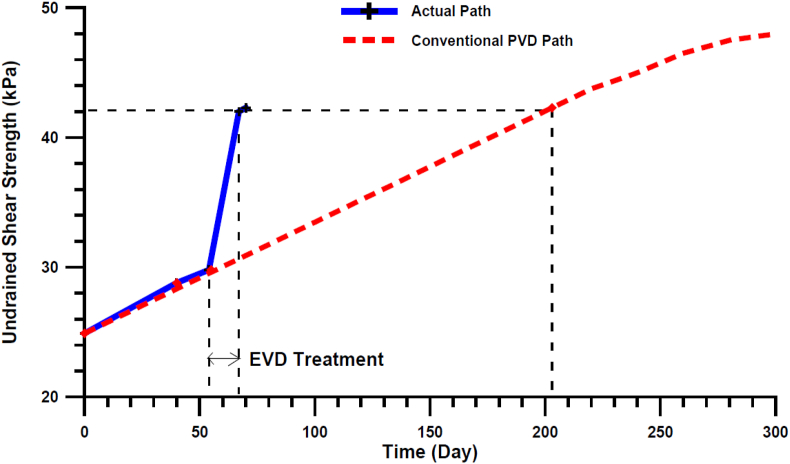
The variation of vane shear strength of soil when electric vertical drains are used compared to prefabricated vertical drains (Chew et al., 2004). Reprinted with permission from Elsevier.

Where k_h_ is the coefficient of permeability, ϒ_w_ is the unit weight of water, and x is the distance between the cathode and anode. Figure 3 show a comparative graph of shear strength of soil when treated with EVD compared to prefabricated vertical drains (Chew et al., 2004).

Field tests were conducted by Ou et al. (2009a) to understand the applicability of the method, with the injection of Calcium Chloride and then Sodium Silicate in soft silty clay. These are conventional chemical stabilisers that could be injected into the soil with pressure and undergoes a cementation reaction. They used nine electrodes of 6.5m length, having the top 1.5m insulated to prevent short-circuiting in 2 m × 2.5 m and 2 m × 5 m arrangement. A thick, cemented, impervious layer of diameter about 50–60mm was formed around the anode. They observed that Ca^2+^ ion in the added solution increase the conductivity of the soil, and its high concentration compresses the double layer that results in the aggregate formation in soil. During the process, temperature increase by 10 °C and 2.5 °C for 2.5 and 5m spacing, respectively, and vane shear strength increased from 15kPa to around 40kPa. Abdullah and Al-Abadi (2010) used a cationic stabilising agent (Ca^2+^ and K^+^) using (1.0M Ca(OH)_2_, 1.0M CaCl_2_, 1.0M KOH, and 1.0M KCl) and found K^+^ more effective in reducing the plasticity in comparison to Ca^2+^. Plasticity index of soil came down from 40 to 32 and 8, and free swell reduced from 14% to 3% and 0.4% for Ca^2+^ and K^+^, respectively. So, the overall effect is expected to increase the bearing capacity of the soil. Similarly, laboratory experiments conducted by Ou et al. (2009b) on silty clay with CaCl_2_ and Al_2_(SO_4_)_3_ · 18H_2_O and found an increase in shear strength from 10to 70 kPa at the anode with CaCl_2_. Ozkan et al. (1999) deduce a 5 to 6 times increase in shear strength using Phosphate and Aluminium ions.

Ou et al. (2015) identified Sodium Hydroxide, followed by CaCl_2,_ is more effective in increasing cone penetration resistance at higher pH. They conclude that a longer curing time and high potential can significantly increase strength due to the formation of Calcium Silicate/Aluminium Hydrate. Similar tests were carried by Chien et al. (2011) on silty clay, with the injection of CaCl_2_ followed by Sodium Silicate. A hard layer was formed around the anode, and the shear strength of soil reaches around 70kPa against 20kPa for untreated soil. Also, soil settlement was observed as 6.1mm by applying only the electro-osmosis process, which increased to 7.3mm with an injection of CaCl_2_ and further enhanced to 10.8mm with an injection of CaCl_2_ followed by Sodium Silicate. To obtain reasonably uniform cementation rather than improved strength around the vicinity of the electrode, Chien et al. (2010) proposed using a relay pipe at the middle of two electrodes which not only increases strength near the electrode but at the middle portion also. On treating silty clay with CaCl_2_, and Sodium Silicate, the average cone resistance of untreated soil was 180kPa, which increased to 884kPa in case of injection at the anode, and further increase up to 912kPa was observed by using relay pipe. The combined effect of two (injection at anode and relay pipe) at anode brings it to 1616 kPa, which further increases to 3230 kPa with a different injection time combination. A noticeable change occurred in the middle, where strength was 410kPa when chemicals were injected at the anode and increase to 1457 kPa when the relay pipe was used. So, this method could be beneficial for more consistent and extensive improvement in strength. The loading condition before electro-osmosis also affects the final settlement and strength (Jeyakanthan et al., 2011).

While using electrochemical methods, proper care should be taken for a change in pH near the cathode and anode. Mosavat et al. (2012) deduced that these methods are not suitable for soils possessing high carbonate buffer and high cation exchange capacities. Burnotte et al. (2004) observed that the installation cost is higher than the operation cost. However, with other chemicals, higher strength can be achieved, which further increases its effectiveness.

### 2.3 Ground improvement using inorganic pozzolanic/cementitious method

Ground improvement using the inorganic pozzolanic or cementitious method is further subdivided into four types based on the material used for soil improvement.

(a) Cement and lime
(b) Fly ash
(c) Calcium carbide residue
(d) Other inorganic binders such as sodium silicate-based stabilisers

#### 2.3.1 Ground improvement using cement and lime

Cement is one of the most readily available and widely used admixtures for soil stabilisation (Conner and Hoeffner, 1998; Dash and Hussain, 2012). It can significantly increase the strength, durability, and stiffness of weak soil, especially in projects where the cost of excavation for a deep foundation could significantly affect economics. The basic stabilisation principles through calcium-based stabilisers are cation exchange, flocculation, pozzolanic reaction, and cementitious hydration. However, cementitious hydration does not occur in lime. Since cation exchange, flocculation and agglomeration occur within a short duration after application, both cement and lime can reduce the plasticity of soil immediately. Both have their own set of advantages; lime provides a large amount of Ca^2+^ ions, whereas cement can significantly enhance strength and durability by C-S-H bonding. The high pH of the soil also proves beneficial for Calcium-based stabilisers due to the increase in reactivity and solubility of Silica (Prusinski and Bhattacharja, 1999).

Though additions of cement increase the strength of soil, it also increases the brittleness of soil which could lead to catastrophic failure (Jan and Mir, 2018). The humus presence may also pose a problem since such soil's pH is usually low due to humic acid. The most common constituents of organic soil are humic acid, fulvic acid, and humin. Humic acid forms insoluble Calcium humic acid, and fulvic acid inhibits Al, which is a pozzolanic material and form cementitious material like Calcium Aluminium Silicate Hydrate by combining with Aluminium-containing mineral. Calcium and Aluminium Sulphate additives can be used to prevent the above reactions. Excess of Ca^2+^ prevents precipitation due to humic acid, and Aluminium Sulfate compensates for the loss of Aluminium mineral due to fulvic acid (Chen and Wang, 2006). Stabilisation with cement is less dependent on soil properties compare to lime since cement contains silica, unlike lime which reacts with silica from broken clay particle. Nevertheless, care should be taken while using both materials since both can undergo carbonation by reacting with carbon dioxide from the atmosphere (Asgari et al., 2015).

Lime and Cement columns could be used if mixing or transporting a large soil volume is not feasible. The strength and rate of reaction of columns depend on the temperature and pH of the soil. The shear strength of such a column is not uniform but sometimes higher than the laboratory determined value. Broms (1991) deduced such columns' utility in reducing settlements (both total and differential) and vibrations in soil due to dynamic loading. Ayeldeen et al. (2016) highlighted the importance of avoiding the cement-soil mixture's storage before compaction. Since cementation starts immediately, disturbed samples offer much lesser strength compared to the undisturbed sample. da Fonseca et al. (2009), while working with fine and residual sand mixed with cement and deduced following relationship between UCS (q_u_) of the soil and the ratio of void volume (V_v_) to the volume of cement (V_ce_):

(3) $${\text{q}}_{\text{u}}\left(\text{kPa}\right)=\text{A}{\left[{\text{V}}_{\text{v}}/{\left({\text{V}}_{\text{ce}}\right)}^{\text{c}}\right]}^{-\text{b}}$$

Where A, b, and c are constants depending on the type of soil and cement. Akpokodje (1985), while working with sandy loam and clayey loam, identified a substantial, almost linear increase in UCS when treated with cement and lime. However, this improvement was not evident in highly gypsiferous/bassanitic soil. Umesha et al. (2009) worked with dispersive soil and concluded that lime could be effectively used for reducing dispersivity. Simultaneously, dispersion drop to 35% from 50%, a slight increase in liquid and plastic limit was also observed up to 1% of lime. After that, no significant change was observed. Their study showed that lime could be used to reduce the erodibility of soil.

Rice Husk Ash (RHA) and Ground Granulated Blast Furnace Slag (GGBS) have been used as a partial replacement for cement due to the harmful effect of cement production (release of CO_2_, dust, and a large amount of energy consumption) (Paul and Hussain, 2020). Maximum dry density (MDD) increase and optimum moisture content (OMC) decrease with the addition of cement, and a similar trend was followed with partial replacement of cement with GGBS and RHA up to 30%, but after which MDD decrease and OMC increase. UCS of cement replaced treated soil was lesser compare to pure cement mixed soil, but for soil with low organic content, 30–50% cement can be replaced with GGBS or RHA. The pH of soil also reduces with the replacement of cement with GGBS and RHA due to the lesser supply of Ca^2+^ ions. Similarly, Liu et al. (2019b) used a composite of cement, steel slag, and metakaolin for soft clay, and it was evident that bind/absorbed water by clay minerals does not take part in the hydration process. With the addition of composite, UCS of soil increase significantly, but with an increase in water content, UCS decrease due to enlargement of the cluster resulting in weaker soil fabric and cementation. They proposed the strength of soil was proportional to binder-free water ratio (C_s_/W_f_) where C_s_ is the amount of binder and W_f_ is free water content which can be expressed empirically as W_f_ = water content – n∗plastic limit, n depends on curing period of soil. Metakaolin is a Calcium-based stabiliser, and it is produced by calcination of kaolinite at a temperature above 500 °C. An Alumino-Silicate compound usually used with cement to enhance the UCS and tensile strength of soil (Behnood, 2018).

Jha and Sivapullaiah (2015) analysed the stabilisation mechanism for clays having high compressibility and swelling properties by using lime, focusing on the structure of the soil matrix. With the addition of lime, flocculation and cation exchange takes place immediately, which affect the atterberg limit. However, since this flocculated structure is not very strong, so compressibility of soil did not change appreciably. Cementation takes time to occur, and the voids are filled with cementitious gel. They also conclude that at higher concentrations, cementation dominates, whereas, at lower concentrations, flocculation and cation exchange occurs. Thus, the permeability of soil first increases due to the aggregation of particles and then decreases over time due to the filling of voids. Cementation and aggregation may take a year-long time to complete, and strength keeps increasing (Arabani and Karami, 2007). According to Hussain and Dash (2010), a small quantity of lime reduces MDD and increase OMC, while more than 5% addition of lime increases MDD and reduce OMC.

Osuolale et al. (2017), working on laterite soil and cement mix, deduced that both bearing capacity and strength (CBR and UCS) decrease with delay in compaction. The additional energy needed to break lumps and bonds formed in soil may be the reason for the loss in strength. Similarly, Raja and Thyagaraj (2020) found a decrease in MDD with compaction delay on clay and lime mixture, and a significant reduction was observed during the first 6 h. They also proposed the use of Sulfate solution to inhibit the formation of aggregate. A concentration of 20000 ppm Sulfate solution is effective in retarding the rate of reaction but significant risk of formation of immediate ettringite on the addition of cementitious stabilisers, which could further reduce the MMD achieved.

#### 2.3.2 Ground improvement using fly ash

Fly ash (FA) is an industrial waste generated by the thermal power plant. India produces around 112Mn ton of fly ash every year, whereas more than 500Mn ton of fly ash is produced per year worldwide (Ahmaruzzaman, 2010; Dwivedi and Jain, 2014). Engineers utilise this waste material as a soil stabiliser since the early 20th century due to its vast potential in binding capacity and availability at low cost (Jacobs and Bennett, 1950). Generally, fly ash is classified into Class C and Class F depending on the availability of cementation agent. Class C fly ash contains a high amount of Calcium Oxide and possesses excellent cementation properties. In contrast, Class F fly ash does not possess much cementation properties due to less Calcium content (Misra, 1998; Arora and Aydilek, 2005). In the latter case, an activator such as lime or cement is required to improve the cementation property.

The pH of the soil also plays a vital role in governing the account of leaching (Sauer et al., 2012). This can be attributed to the fact that the pH of soil regulates the solubility of the ions. Tastan et al. (2011), while studying the stabilisation of organic soil, deduced a careful utilisation of fly ash since organic soils are problematic due to humic acid and high-water absorbing capacity, which left much lesser water for hydration. It may be possible that the calcium ions of fly ash are not available for the reaction because it may be absorbed, precipitated, or formed a complex compound with soil. Zha et al. (2008), in their study on expansive soil, deduced that the addition of fly ash could immediately decrease plasticity and swelling due to cation exchange reaction (due to the presence of Ca^2+^, Al^3+^, Fe^3+^). The addition of fly ash decreases the thickness of the diffused double layer, causing flocculation and an increase of coarser particles in the soil, thereby decreasing the density (generally, but depends on soil). It was found that the pozzolanic reaction dominates over cation exchange and incorporation of lime further strengthens the soil by breaking montmorillonite. Lime provides more Ca^2+^ for reaction with silica and alumina to form a hydrate of Calcium Silicate and Calcium Aluminate. Also, the acid present in the soil gets neutralise by lime, thus providing optimum pH for reaction (Sharma et al., 2012). Joseph et al. (2018) concluded that a vertical fly ash column could reduce the swelling by 44.5%. A mixture of lime and fly ash can be used effectively in silty soil possessing lower plasticity. In their experiment, they found the optimum ratio of lime: fly ash as 1:4 for cochin marine clay.

**Figure 4:**
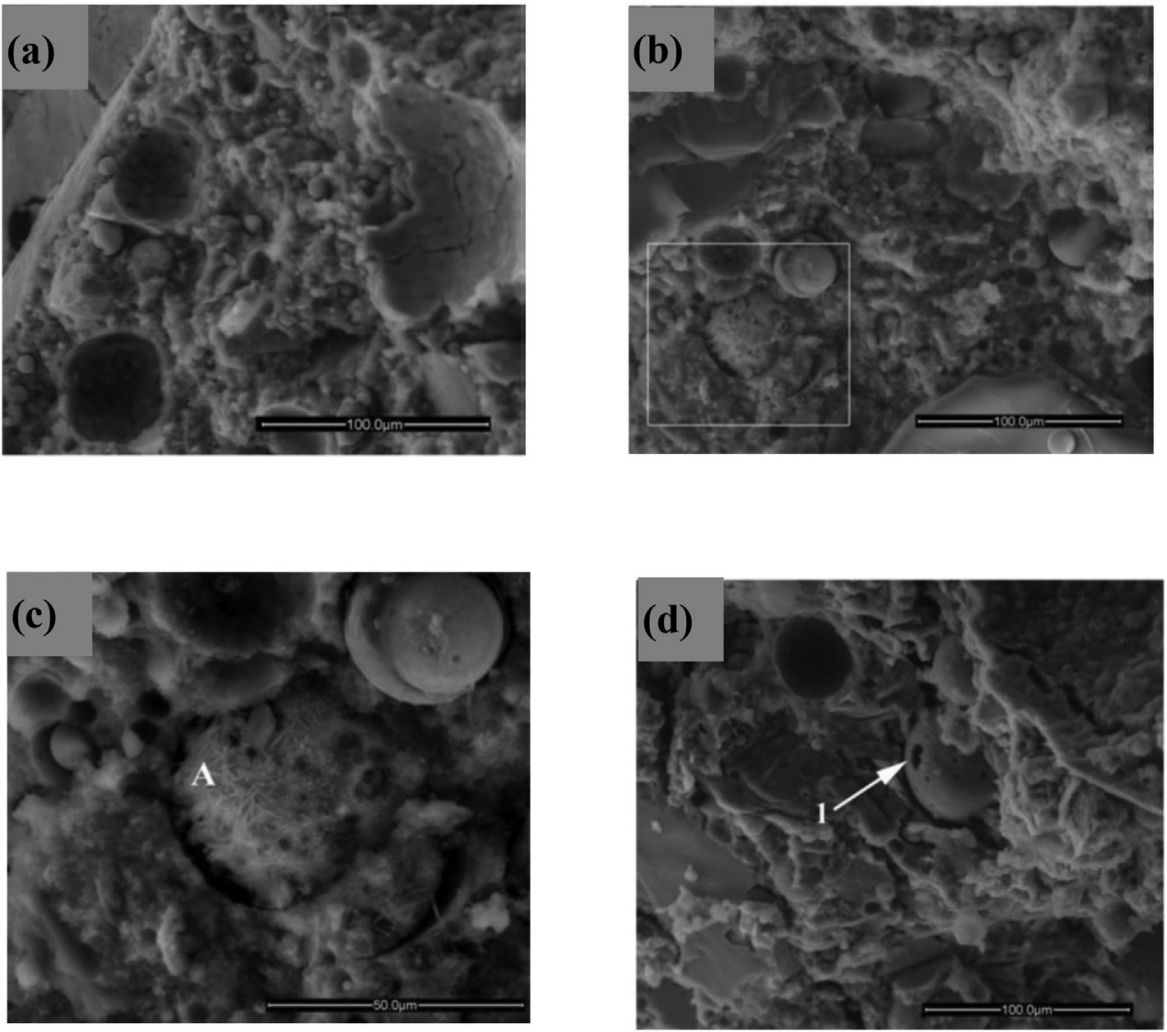
SEM images of soil fly ash mixture after 1 (a), 3 (b and c) and 7 (d) days curing (Cristelo et al., 2012). Reprinted with permission from Elsevier.

The Shear strength (cohesion and friction angle) can be increased with fly ash application (Hasan, 2012; Consoli et al., 2001). The increase in cohesion is due to the cementation reaction shown in Figure 4 (Cristelo et al., 2012). The increase in friction angle is due to a change in texture during flocculation. Prabakar et al. (2004) concluded that the addition of fly ash in highly cohesive soil brings down the cohesion while increasing the angle of friction. According to Sridharan et al. (1997), this can be attributed to the silty nature of fly ash which was later validated by Rajak et al. (2019). Bin-Shafique et al. (2010) and Karim et al. (2017) studied the effect of fly ash on the durability of plastic and expansive clay and concluded that UCS of such soil increase with cycles of wetting while decrease with a freeze-thaw cycle which could occur due to expansion of water in pores. Hardaha et al. (2015) emphasised the use of fly ash in controlling the swelling behaviour of black cotton soil. They observed a significant decrease in the free swell index (FSI) from 66.6% to 4.2%, with 0% (w/w) to 50% (w/w) of fly ash. Results also show improvement in the plasticity of the soil, changing from CH to MH.

Mackiewicz and Ferguson (2005) studied the utility of using sub-bituminous coal ash and identified a higher degree of cementing characteristics owing to the presence of a higher amount of Calcium (20%–30%) in such ash. According to Kalita and Singh (2010), about 20–25% of Class F fly ash was sufficient for significantly improving strength. However, some amount of cement is also required as it lacks cementation properties for its use as the upper layer of roads.

The compaction properties of soil can be changed due to the presence of coal ash. It was observed that the OMC for maximum strength occurs at 1%–7% below MDD. They further analysed the moisture density relationship and concluded that with delay (after fly ash incorporation), maximum density achieved decreased, reducing maximum compressive strength. This happens because as soon as fly ash was mixed, the cementation process starts, and a fraction of compaction energy goes into breaking the bond. A decrease of 0.6–1.6 kN/m^3^ in maximum density was observed with a delay of 1 h. This delay depends on the hydration rate of fly ash and varies for fly ash from a different source. Pandian and Krishna (2003) mixed fly ash with black cotton soil in different proportion of weight. They noted that most of the cementation occurs within the first week of addition, and a decrease in both OMC and MDD was observed with an increase in the percentage of fly ash.

Researchers also utilised rice husk ash and coconut husk ash for ground improvement and found similar results (Amu et al., 2011; Oluremi et al., 2012; Ambarakonda et al., 2019; Rathan Raj et al., 2016). Muthukkumaran and Selvan (2020) deduced a decrease in the liquid limit, plastic limit, and shrinkage limit of soft soil owing to the presence of Ca and Mg in neem leaves ash. The FSI came down from more than 800% to less than 200%, with 10% neem ash. However, there was not a clear trend in the variation of UCS. Rao et al. (2000) used wood ash in black cotton soil and found a similar result when lime is used along with ash. Sivapullaiah et al. (2004) used lime mixed with rice husk ash as a cushion between the foundation and black cotton soil since ash alone was insufficient to produce the desired stabilisation. They found lime mixed ash more effective to stabilise foundation with 6% of lime and one week curing period.

#### 2.3.3 Ground improvement using calcium carbide residue

**Figure 5:**
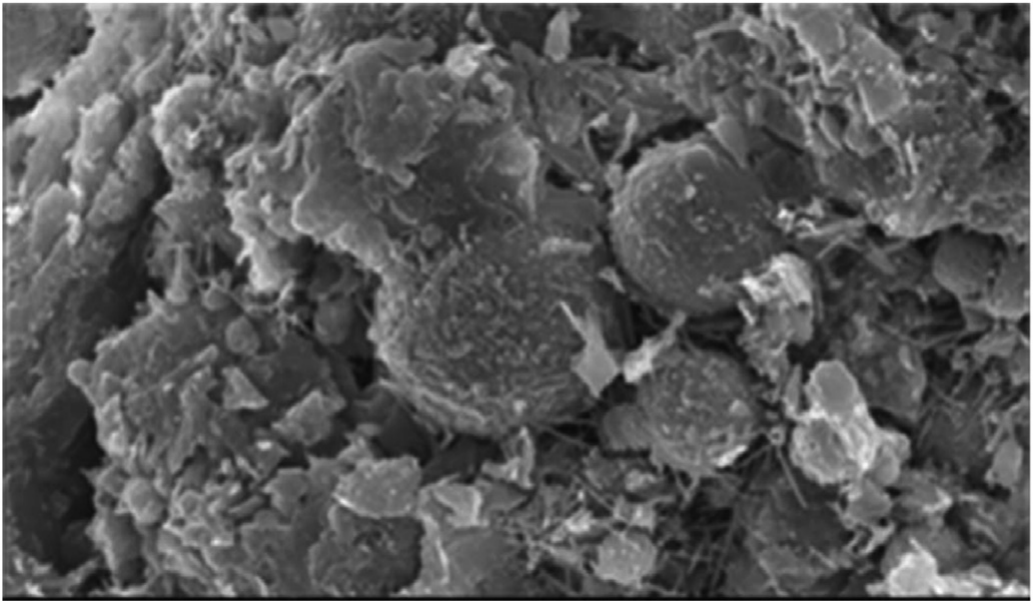
CCR stabilised clay (Kampala et al., 2013). Reprinted with permission from Elsevier.

Calcium carbide residue (CCR) is produced as a byproduct during the production of acetylene. Like fly ash, its use as a stabiliser is also a good solution for its disposal. It helps in reducing swelling and increasing the friction angle of the soil (Juneja and Shinde, 2019). Calcium Hydroxide (Ca(OH)_2_) is the main constituent of CCR, along with some minor concentrations of Calcium Carbonate. Though it is not dangerous for disposal purposes, the precaution should be taken due to its high alkalinity. Sometimes the availability of heavy metals cannot be ruled out. Due to the high concentration of Ca(OH)_2_, it is suitable for clayey soil as they already contain a sufficient amount of pozzolanic materials such as Ca and Al. Ca(OH)_2_ reacts with these minerals to form cementing compounds, as shown in Figure 5 (Kampala et al., 2013). Many times, it is proved to be more effective than lime stabilisation (Kampala and Horpibulsuk, 2013). Latifi et al. (2018) conducted several UCS tests with clay and CCR mix and deduced a substantial increase in strength property from untreated clay to treated clay along with curing for the initial 28 days. With an increasing amount of CCR, the soil starts developing favourable properties that become constant after a certain amount; it is called optimum CCR concentration.

Du et al. (2016) performed field experiments with CCR and deduced an almost similar compaction curve of that of lime stabilisation on the dry side. However, the stabilised soil had more MDD on the wet side and nearly the same OMC with 0.1% variation. The CBR value for CCR was also found higher than lime stabilised soil, but a more extended curing period was needed in the case of CCR. Similarly, a higher value of resilient modules and lower resilient deflection was noticed. Similar results were found by Kumrawat and Ahirwar (2014) by mixing CCR and stone dust with black cotton soil. The mixing of stone dust further enhances the workability of soil. According to Latifi and Meehan (2017), bentonite and kaolin show a decrease in MDD due to the lower density of CCR, and an increase in OMC may be due to the higher specific surface area.

Kumpala et al. (2012) highlighted the importance of optimum CCR concentration, which can be easily determined by consistency limits. The initial increase in CCR content improves the soil by cation exchange and reducing the double layer thickness to optimum concentration. Increasing the concentration after the optimum level does not produce any visible improvement of soil. With increasing moisture content, repulsive forces in clay also increase, but moisture also supports pozzolanic reaction. OMC provides sufficient moisture for the development of strength without increasing repulsive forces much. Joel and Edeh (2013) showed statically with the Chi-square (χ^2^) test that the properties of lime and CCR stabilised laterite soils are nearly the same. Horpibulsuk et al. (2012) advocated using CCR along with fly ash due to the similarity in the mechanism of stabilisation and both being industrial waste products. Fly ash contains a high amount of pozzolanic materials, while CCR has a high percentage of Ca(OH)_2_, which provides Ca for reaction in the case of class F fly ash. Naveena et al. (2017) proposed a regression equation for the UCS of CCR treated soil:

(4) $$\text{UCS}=\left[\left\{-370{\left(\frac{{\text{W}}_{\text{c}}}{CCR}\right)}^{2}-510\left(\frac{{\text{W}}_{\text{c}}}{CCR}\right)+300\right\}\left\{-0.0707\left(\frac{D}{{\text{I}}_{\text{p}}}\right)\right\}\right]-4.\text{ CCR}+\text{E}.\text{BS}$$

Where Wc/CCR is clay-water to CCR ratio, D is curing period (days), CCR is in percentage, Ip is plasticity index, BS is UCS at zero CCR content, and E is a constant which depends on BS.

Soil stabilised with CCR can be classified into three parts, namely, active zone, when the addition of CCR increases strength; inert zone, when the addition of CCR does not alter strength properties significantly; and lastly, the deterioration zone when the addition of CCR reduces strength. The addition of fly ash in the active zone is not useful since plenty of natural pozzolanic materials are already present. However, adding in the inert zone can increase strength multiple times. The increase in strength in the deterioration zone with the addition of fly ash is low compared to the inert zone due to excess Calcium, and also, it is not economical to use fly ash in the deterioration zone (Horpibulsuk et al. 2012, 2013). Kampala et al. (2014) found that the incorporation of fly ash also useful to increase the durability of soil; after multiple wet-dry cycles, the performance of FA + CCR mixed soil was better. They found 7% CCR and 20% FA as optimal content. Similar results were also found with coconut ash and biomass ash (Vichana et al., 2013; Vichan and Rachan, 2013; Isahand Sharmila, 2015).

#### 2.3.4 Ground improvement using other inorganic chemicals

Many environmentally friendly and safe inorganic chemical compounds such as Silicate-based chemicals and some Mg, Ca based salts are also used as soil stabiliser. They usually react with soil to form strong bonding between particles or themselves get harden. Calcium Silicate Hydrate, which is the main product of cement, forms after the hydration reaction and is responsible for the settlement of cement. Not only in cement but also lime and fly ash-based stabilisation, the formation of calcium silicate is of critical importance (Bullard et al., 2011). Many times, Sodium Silicate and Calcium-based compounds are directly injected into the soil, which results in an inorganic polymeric compound, Calcium Silicate Hydrate. Huat et al. (2011) identified Sodium Silicate (or Sodium metasilicate) as one of such traditional additives that is eco-friendly and can be used with cement. Its other form, such as Sodium Orthosilicate and Sodium Sesquisilicate, when added alone, do not give satisfactory results. However, their application, when added with other stabilisers such as lime or fly ash, shows promising results (Rafalko et al., 2007). Madurwar et al. (2013) deduced that the possible reason might be its solubility. Hurley and Thornburn (1971) identified the injection of Sodium Silicate followed by Calcium Chloride results in insoluble gel formation, which fills up the voids in the ground.

The alkalinity of the soil increases with an increase in Sodium Silicate, which helps in increasing the strength of the soil. When used with lime, it produces Calcium Silicate, which also increases strength and reduces swelling of soil (Maaitah, 2012). Due to the low solubility of Sodium Silicate and lime mixture, it is difficult to use them for in situ grouting. For in situ grouting, Ma et al. (2015) modified the mixture by mixing Sodium Silicate with cement and some promoters. Apart from ordinary portland cement and Sodium Silicate, NaOH was used to increase pH, Ca(OH)_2_ for promoting cementation reaction, Na_2_Al_2_O_4_ for producing mineral gel, and anhydrous CaCl_2_ was used as an accelerator. Moayedi et al. (2012) found an increase in UCS due to promoters (CaCl_2_ and Al_2_(SO4)_3_) was more pronounced until 0.1M, after which its effectiveness decrease. Using 3M Na_2_Si_2_O_3_ and 0.1M activator, about 270 % increase in UCS was observed. Latifi et al. (2014), while working with laterite soil, concluded that the compressive strength starts decreasing by increasing the percentage of sodium silicate-based stabiliser beyond 9%. Gobinath et al. (2020) observed a considerable increase of about five times in UCS and more than ten times in CBR for gravelly sand when treated with 1% Sodium Silicate and 0.5% banana fibre. Due to the absence of Ca in Sodium Silicate-based stabilisers, Pakir et al. (2015) observed that it could apply to Sulfate-containing soils, which are otherwise problematic with traditional stabilisers such as lime, cement, and fly ash. If one still wants to use Calcium-based stabilisers such as Sodium silicate with Calcium Chloride, it is advised to pre-treat soil with Barium which forms an insoluble precipitate with Sulfate preventing further reaction (Kota et al., 1996).

Chloride compounds are also used as soil stabilisers, but they have their advantage and disadvantages. Afrin (2017) conducted various tests on clayey soil using NaCl, CaCl_2,_ and MgCl_2_and observed a slight increase in MDD without any considerable OMC change for all three salts up to 12% concentration. This was attributed to the filling of void spaces by the salt. Atterberg limits also show a declining trend with an increasing percentage of salt. A similar decrease in swelling index was noticed, with NaCl being the most effective salt, while MgCl_2_ was the least effective. Abood and Mohamed (2014) also deduced similar trends; however, they found a decrease in OMC. About 200–300kPa increase in UCS was observed with increasing salt concentration up to 8%. Krishna and Ramesh (2012) obtained an increase in bearing capacity from 1360 kPa to 5198 kPa with a 3% addition of CaCl_2_. This is attributed to soil particles' clustering when Calcium Chloride is added to the soil along with water. Shon et al. (2010) used fly ash and CaCl_2_ together, exploring the synergic effect of the two. Adding CaCl_2_ can fulfil the deficiency of Ca in Class F fly ash. They found a slight increase in OMC value which is due to the hygroscopic nature of CaCl_2_. This effect was the same for both Class C and F fly ash. Soil sample treated with 1.7% CaCl_2_ and 10% fly ash showed the highest UCS with more than a 20-fold increase in the case of both Class F and C fly ash. However, for Class C fly ash, other compositions do not show such an enormous increase in strength over the entire period. The addition of CaCl_2_ in a dry environment shows fast development of early strength but in the case of a wet environment, chances of leaching increases in the early days.

Similarly, Ghavami et al. (2020) explored the synergic effect of cement kiln dust and NaCl on kaolinite clay and found similar results. Thyagaraj et al. (2012) utilised CaCl_2_ and NaOH for in situ Calcite precipitation and found mixing or injection of NaOH after CaCl_2_ more effective than vice versa. Added NaOH will also contribute to the cementation reaction between Ca and Silica. This method may help repair work beneath the foundation due to the reasonably good solubility of the two reagents. Other chemicals such as Sodium Carbonate, Calcium Carbonate, and Sodium Hydroxide can also be used for improving the properties of soil. Calcium Carbonate is more effective than Sodium Carbonate due to the presence of Ca^2+^ ion, while Sodium Hydroxide is more effective on Al-containing soils (Ramesh et al., 2012; Olaniyan et al., 2011).

Silica fumes are another non-crystalline industrial waste product mainly from electric arc furnace during the production of silicon or silicon-containing ore like Iron; since Iron contains a good quantity of Silica, most steel plants also produce silica fumes in large quantity. About 100000 tons of Silica fumes produced annually worldwide. Its use as a ground stabiliser is a good option for both construction purposes and waste utilisation. It increases the bond between soil particles, so an increase in cohesion is observed, resulting in increased UCS and CBR value of foundation soil. It contains mainly silica (SiO_2_) and other oxides of Iron, Aluminium, Magnesium, Calcium and Sulphur in minor fraction (Gupta and Sharma, 2014; Singh et al., 2020). They are mostly suitable for expansive soils and decrease soil permeability (Sabat and Pati, 2014).

### 2.4 Ground improvement using organic polymeric binders

There is major four organic polymeric that can be used as binders for soil improvement.

(a) Polyurethane
(b) Lignosulfonate
(c) Epoxy resins
(d) Polyacrylamide

#### 2.4.1 Ground improvement using polyurethane

Polyurethane (PU) is a polymer made up of Polyol (-OH) and Polyisocyanates (-NCO). Different types of Polyol and Polyisocyanates result in a different variety of polyurethane. Its rapid reaction time (usually between 30 to 120 s) and lightweight make it suitable for repairing highway pavement since it cannot be blocked for a long time. Groundwater or the presence of moisture in soil could be a limiting parameter for the use of polyurethane since the liberation of gas, which could be CO_2_ due to the reaction between water and isocyanate or water vapour due to heat from the reaction of Polyol and Polyisocyanates (Yu et al., 2013). Some catalysts, chain extenders, and surfactants are also used to get desirable properties (Saleh et al., 2019). The moisture content of soil also regulates the density and yield of a reaction. Usually, PU is viscous and is suitable for medium-size sand, but recent developments are underway to make it suitable for silty and clayey soil (Robinson et al., 2012). Hydrophilic PU can accommodate a large quantity of water in their structure, due to which their volume increases several times. They are commonly used for sealing cracks or ground improvement where strata bear a large amount of water (Vipulanandan et al., 2012). With the injection of PU grout, the expansion of polyurethane grout displaces water and soil gets consolidated. Also, most of the stabilisation occurs within 15 min of application (Fakhar and Asmaniza, 2016).

**Figure 6:**
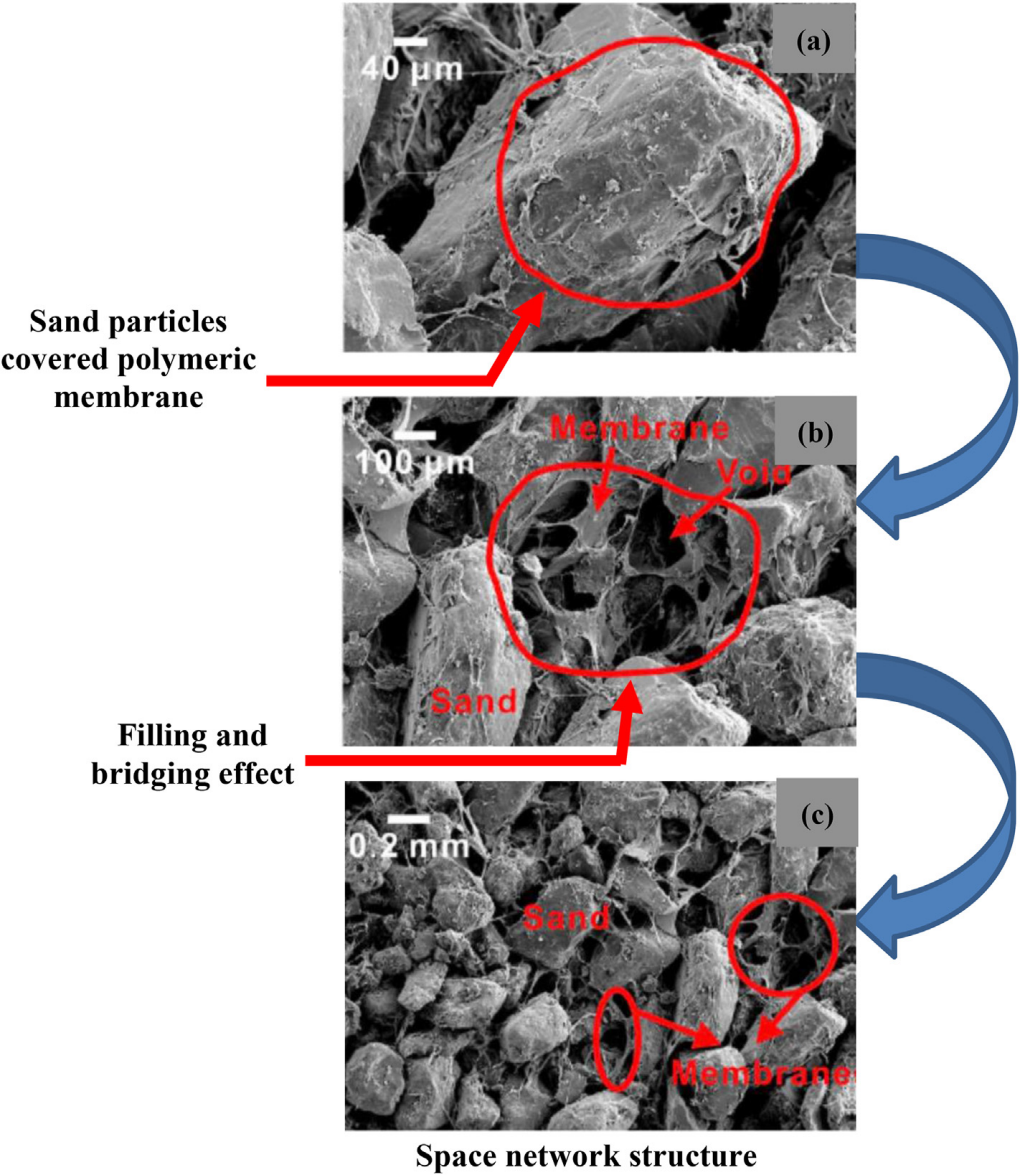
SEM image of PU treated sand (Liu et al., 2019a, 2019b). Image (a) shows the closed enwrapping of sand particles by a fine hard coating of polymeric membranes. Image (b) shows the supplementary improvement of cementation via a chain networking of the interrelated membranes due to growth of the inter-particle voids filling. Image (c) shows the structure completion of the 3D cross-linked network to a huge degree. Reprinted with permission from Elsevier.

PU immediately increases the bearing capacity of the soil and is a suitable stabiliser for damaged foundation soil (Sabri et al., 2018). Liu et al. (2018) studied the effect of polyurethane mixed with polypropylene fibre on the tensile strength of sands. They found that curing of at least 12 h is required for solidification of the specimen and about 48 h for stabilisation. In the study, they showed that polyurethane helps in binding polypropylene with soil particles resulting in greater tensile strength, which could be 2 to 3 times initial tensile strength depending on the compaction. Liu et al. (2012) showed that polyurethane can significantly increase soil resistance towards erosion and could be beneficial for clay-rich soils. According to Liu et al. (2019a), traditional methods of stabilisation (like cement and lime) can improve engineering properties effectively. However, its use can increase the brittleness of soil which could lead to sudden failure of structure and also have environmental issues. They used water-based polyurethane stabilisers (activate in the presence of water), with different concentrations ranging from 0 to 40%, and obtained a substantial increase in cohesion, friction angle, and tensile strength for 40% PU. Similarly, hydraulic conductivity decreased from 1.43 × 10^−2^ to 7.23 × 10^−4^ cm/s for a PU concentration of 3–5%, making the soil stable against water. Liu et al. (2019a) also highlighted the negative impact of using PU in the soil since filling PU in voids of soil renders the growth of vegetation as roots cannot expand freely in such soils. This is shown in Figure 6 (Liu et al., 2019a).

Komurlu and Kesimal (2015) showed that rock-like strength could be achieved by polyurethane soil mixture without significantly increasing the weight of soil. With a 1:7 ratio of PU:Soil by weight, they achieved a much durable UCS sample of more than 4MPa with sandy silt. Saleh et al. (2018) found that with the addition of 0–5% PU in marine clay of Malaysia, maximum deviator stress increased up to 501kPa from 150kPa. With an increase in the percentage of PU, a decrease in the deformation of the sample was also observed. Zhang et al. (2019) studied the cracking and shrinkage behaviour of bentonite clay with polyurethane and found an increase in stiffness of the sample. Hydrophilic PU was more effective in controlling shrinkage distress.

Keene et al. (2013) found PU more suitable for coarser material since it could lead to hydro-fracturing in fine-grain clayey soil at the point of injection due to its viscosity. It was found that there was a significant increase in plastic strength under cyclic loading. At the same time, a decrease in the elastic property was also observed. Thus, there is a need for analysing its overall effect. The average resilient modulus decreases to 100MPa from 275MPa. Xiao et al. (2018) studied well-graded angular and subangular gravel stabilised with PU and concluded that with the addition of PU, there is increase cohesion without any reduction in angle of friction, unlike in some cases of fly ash. Compared to cement and lime-based stabilisers, it does not increase the brittleness and incorporate good post-failure strength. With 0–8% addition of hydrophobic PU and curing for 60 min, brittleness index [(peak deviatoric stress/residual deviatoric stress)-1] for 0.5MPa confining pressure varied from 0.01 to 0.29 whereas, in the case of lime/cemented gravel, it varied from 0.03 to 2.36. It was also noted that the failed specimen was intact after failure, and their behaviour changes from contractive to dilative with the addition of PU.

Zhou et al. (2018) studied the effect of various parameters like temperature over PU modified specimen and deduced that with an increase in temperature, the strength of specimen increases and then becomes constant after a specific temperature. This is due to a reduction in viscosity of PU, which makes it occupy more empty spaces of the void. Saleh et al. (2020) studied PU-treated marine clay, and its microstructural analysis showed no pozzolanic reaction or no new compound was formed. The untreated particle had a fuzzy arrangement with cusp like crystal, and after treatment with 8% PU, the size of the particle decreases, which increase the density of soil and led to an increase in shear strength and reduction in axial strain. One-dimensional swelling and compression index changed from 0.2682 and 0.0049 to 0.0061 and 0.0017, respectively with 4%–12% of PU.

#### 2.4.2 Ground improvement using lignosulfonate

**Figure 7:**
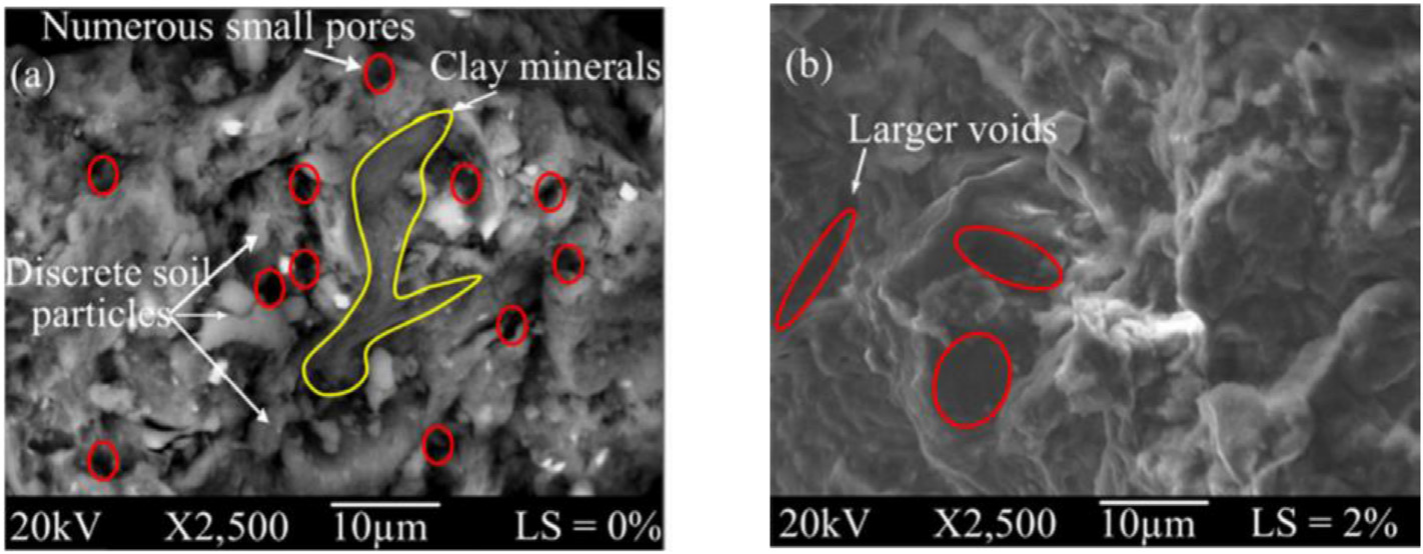
Treated and expansive untreated soil (a) LS 0 % (b) LS 2% (Alazigha et al., 2018). Reprinted with permission from Elsevier.

Lignosulfonate (LS) is an environmentally friendly waste product generated from wood and paper industries having excellent soil improvement application, usually for cohesive expansive soil where the change in chemical composition is not a big issue. It is a cross-linked lignin-based amorphous polymer with a net negative charge, forming a metal ion coordination bond. When mixed with soil, it compacts soil and increases amorphousity, thereby reducing the water-absorbing capacity. The SEM images shown in Figure 7 indicates an increase in the density of soil after the use of LS (Alazigha et al., 2018; Indraratna et al., 2012). It does not increase the pH and brittleness of soil, unlike cement and lime-based stabilisers, which could be dangerous during dynamic and impact loading (Chen and Indraratna, 2015). Vinod et al. (2010) concluded that the addition of Lignosulfonate in soil reduces the coefficient of soil erosion and double layer thickness of clay. Lignosulfonate can be effectively used during construction since it does not pose any threat of sulphate attack on steel or cement structure or problem for vegetation or groundwater contamination due to high pH.

Lignosulfonate has a great potential to increase the resistance of soil against erosion by improving tensile strength at comparably lesser quantity than other admixtures (Vinod and Indraratna, 2011). It creates a polymer chain with soil aggregate, as shown in Figure 7 (Alazigha et al., 2018) and prevents the water flow from dislodging the particles. Koohpeyma et al. (2013) performed a hole erosion test on clayey sand with 3% of LS and deduced that the soil classification changed from extremely rapid to moderately slow erodible soil while the coefficient of soil erosion increased from 0.000017 to 0.01020. Simultaneous use of LS and polypropylene could improve the strength and erodibility of soil. A mix of 0.35% polypropylene fibre and 2% of LS was found optimum to achieve erosion resistance (very rapid to extremely slow) and strength improvement (about 5.5 times). Polypropylene fibre prevents cracking, and LS suppress electrostatic repulsion between clay particles. Chen et al. (2014) found that 2% LS is optimum for sandy silt without ductility loss. Vakili et al. (2018) studied the effect of LS on dispersivity of soil and found 0.5% LS could decrease the percentage of dispersion from 90% to around 40%, with no significant decrease in dispersion with further increase in the percentage of LS was observed. Also, the probability of formation of cracks in soil treated with LS gets highly reduce due to an increase in adhesion and ductility of soil by LS.

Sezer et al. (2016) investigated properties of bentonite mixed sand with partial replacement of water with LS in different proportions (0.5%, 1%, and 2%) and found the dry side of OMC more effective for stabilisation and is suitable for reducing the brittleness of soil. It is more beneficial for the clay of low plasticity. Ding et al. (2018), while investigating dust generation from red sand stabilised with Sodium and Calcium LS, found Sodium LS to be more productive. With an increase in the concentration of LS, the water holding capacity of soil increases, which reduces dust generation. Penetration resistance obtained from the fall cone method indicates an increase from 3.15N to 9.61N, whereas UCS increases more than three times by adding 10% of LS. It was also observed that the viscosity of the water-LS solution increases with an increase in the percentage of LS; thus, the thickness of the stabilised layer decreases with an increase in LS%. Noorzad and Ta'negonbadi (2018), in their study, deduced a slight decrease in MDD and a small increase in OMC for expansive clay with increasing LS content. Using Scan Electron Microscope (SEM), they found the formation of soil aggregates, which was initially absent, and soil was flaky and discontinuous. Whereas Ravishankar et al. (2017) concluded a decrease in OMC and increase in MDD with an increase in LS content and increase in UCS for Lateritic soil but sample failed to sustain wet-dry cycles due to solubility of LS in water.

#### 2.4.3 Ground improvement using epoxy resins

**Figure 8:**
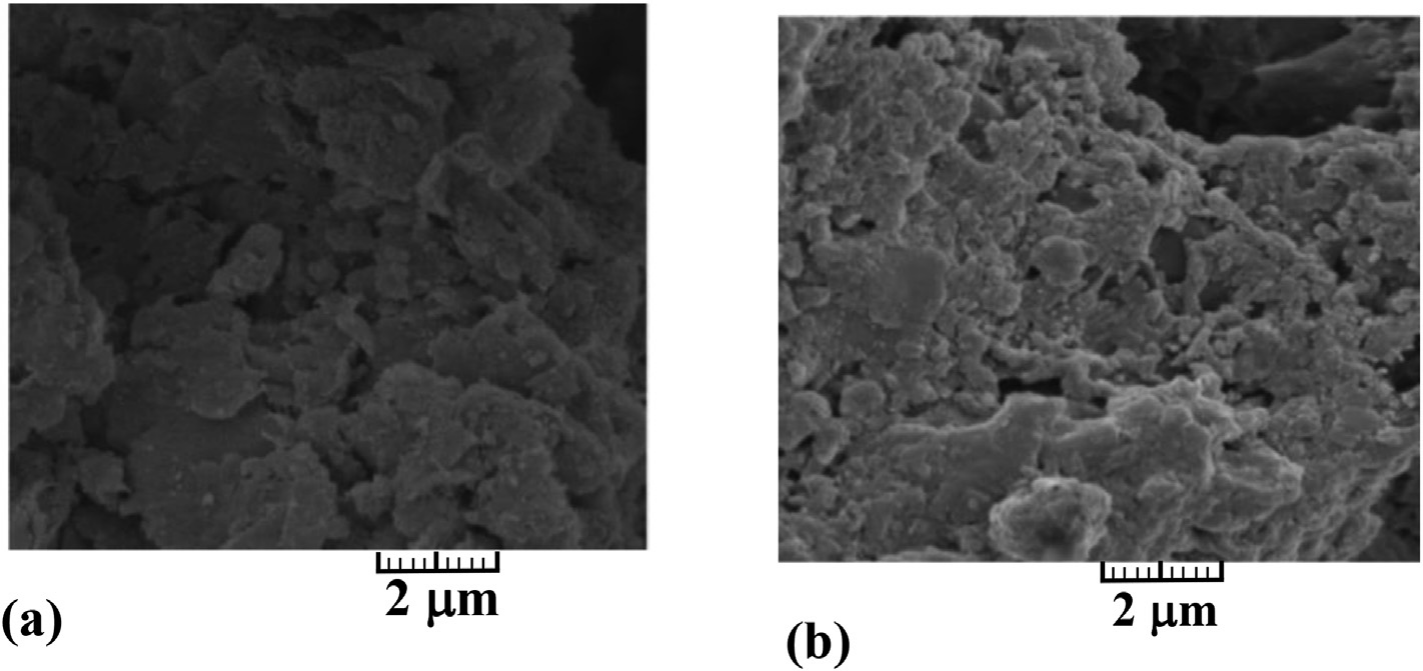
Bentonite treated with epoxy resin (a) bentonite clay (b) bentonite + epoxy resin. Image (a) shows the SEM of the unstable sample. Image (a) shows the SEM of bentonite sample stabilized by the epoxy resin (Hamidi and Marandi, 2018). Reprinted with permission from Elsevier.

Epoxy resins (ER) are liquid pre-polymer possessing excellent tensile and compressive strength. However, they pose a threat to the environment due to their toxic nature (Kazemain and Barghchi, 2012). Epoxy resin undergoes polymerisation reaction when mixed with another compound, usually known as a hardener (epoxy component with amine component). The final product is very stable and resistive against chemicals and heat. It may hinder the pozzolanic reaction in clay stabilised with cement, so it would not be a good idea to use it with cement clay mix. It increases the ductility of soil without reacting with soil minerals (Hamidi and Marandi, 2018), as shown in Figure 8. The application of epoxy resins is relatively superior to cement stabilisation. Due to its high permeability, clogging does not occur at the injection point, and it can be easily used in soil with smaller pores. Water content, curing time, and amount of resin added are significant factors determining the extent of stabilisation. Anagnostopoulos and Papaliangas (2012) studied the effect of dilution on strength and other physical properties of sands and concluded a decrease in UCS and cohesion with decreasing resin/water ratio (ER/W). Resins were good at providing high post-failure strength, inducing strain hardening, but this behaviour was not apparent for highly diluted resins. Consequently, they observed the change in bonding between particles. They also provide a correlation equation for permeability (k) and ER/W.

(5) $$\text{K}\left(\frac{\text{m}}{\text{s}}\right)=4.10^{-5}{\text{e}}^{-5.45\left(\frac{\text{ER}}{\text{W}}\right)}$$

**Figure 9:**
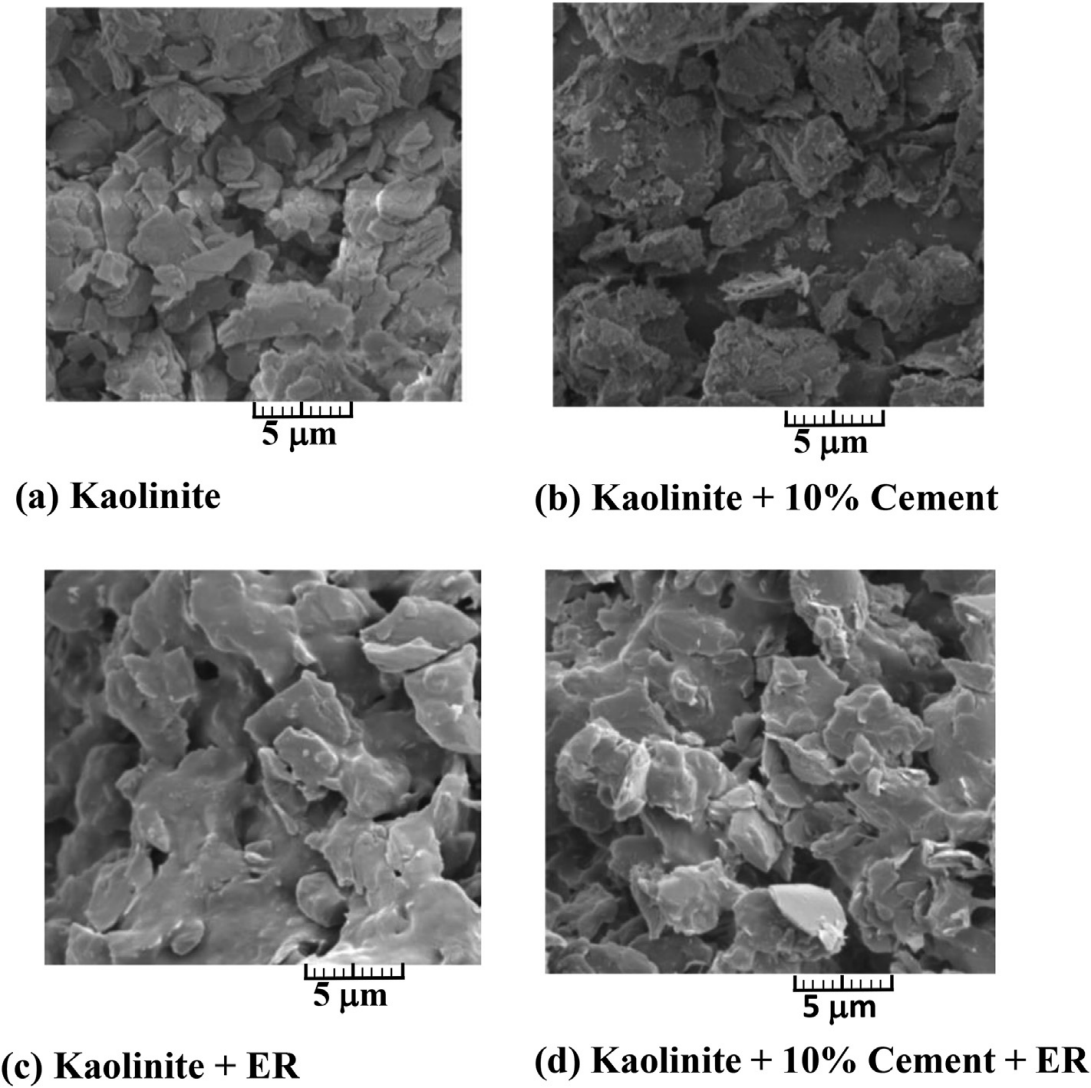
Change in bonding between particles of soil with the addition of ER and cement (Hamidi and Marandi, 2018). Image (a) shows the SEM of the untreated Kaolinite sample. SEM of Image (b) shows the noticeable variations in the structure/morphology of the kaolinite sample stabilized with cement. SEM of Image (c) shows the few variations in the kaolinite structure due to the inclusion of epoxy resin. Image (c) shows the SEM of the Kaolinite sample stabilized by addition of epoxy resin and 10% cement. Reprinted with permission from Elsevier.

Similarly, Anagnostopoulos et al. (2014) investigated the effect of epoxy resin with electro-osmosis and found a further enhancement in the strength properties of sand. Apart from decreasing strength with more water content, time is taken by sample for curing also decrease. Modulus of elasticity decreased from 585MPa to 155MPa for ER/W ratio 2 to 0.5. Usually, the addition of resin results in an increase in MDD and decrease in OMC, but still, these properties are more dependent on soil composition (Ateş, 2013; Kumar and Kumar, 2015). Anagnostopoulos (2015) used epoxy resins with cement to stabilise silty clay and found it useful and reduced brittleness, unlike other soil cement mix. SEM images showing the effect of ER with soil and cement is shown in Figure 9 (Hamidi and Marandi, 2018). The use of resins was found useful for reducing the dispersivity of clays by Vyas et al. (2011). Toufigh and Rahmannejad (2018) proposed a regression relationship between UCS and curing time (t) for dry sand as:

(6) UCS=a+bt

Where *a* and *b* are constant depending on the amount of epoxy resin and water content.

Toufigh and Rahmannejad (2018) highlighted the negative effect of rain or any events just after the injection of resin since it can lead to a rise in water content, and the desirable stabilisation might not happen. However, once polymerisation occurred, it could withstand many adverse situations firmly. The soil stabilised with resigns can even show higher strength than cement if all conditions are favourable for polymerisation. Similar observations were made by Naeini and Ghorbanalizadeh (2010), where the addition of silts resulted in decreased final strength. This may happen because of the smaller size and large area of finer particles. The use of the same amount of resin cannot cover the full area. Also, silt particles surround sand particles hindering the bond between sand and polymer. The final strength of the sample increase with curing time and decrease in case of the submerged condition. Generally, the effect of wetting diminishes after one week. Currie, Hydendaal and Kenter (1999), in their observation over 11 years, established that rigidity and stiffness of the resin stabilised soil does not show any significant deviation from the initial properties of samples over the decade. For the bearing capacity, Ajayi et al. (1991) propose a regression model: CBR=91.69+11.07(PA)−5.62(PM)+44.97(CS)+0.14(Temp). In this model, PA is epoxy resin additive level (%), PM is moisture content level (%), CS is the clay-silt ratio (decimal), and Temp is the temperature of curing (°F).

#### 2.4.4 Ground improvement using polyacrylamide

Polyacrylamide (PAM) is a water-soluble polymer formed by acrylamide as a monomer unit (Green and Stott, 1999). PAM, which has been used as a stabiliser from the last decade of the 20^th^ century, helps in the aggregation and flocculation of soil and can reduce erosion significantly. It is mainly used for the stabilisation of steep slopes in highways. Aggregated soil provides sufficient void space for the passage of water, thus increasing permeability, which reduces the risk of the development of high pore pressure. Also, the runoff of water reduces, which in turn reduces the erosion of soil. PAM binds the aggregate so when water flows through the soil, and it does not disperse into smaller tiny particles, which generally block the flow. Higher molecular weight PAM is more effective compare to lower molecular weight, and this may be due to longer chain length. PAM is a polyelectrolytic copolymer surrounded by oppositely charged salts. It is also used in agriculture to reduce soil erosion during irrigation. About 2 ppm concentrations can reduce erosion by 85%, but it is highly site-specific. It also acts as a dust suppressor. The addition of salts (e.g. (CaNO_3_)_2_, CaCl_2_, MgSO_4_ & NaCl) certainly increases its effectiveness through site-specificity is not denied. The use of PAM is associated with certain environmental concerns, including its non-biodegradable nature, production of harmful substances during its degradation, and its purity since its monomer is highly toxic and is considered a neurotoxin to humans. However, it is also noteworthy that highly reactive monomer forms free radical that combines with other compounds forms less harmful chemicals and do not accumulate. Its degradation does not result in monomer; instead, bacteria in soil remove nitrogen via deamination (Orts et al., 2007). Biodegradation, photodegradation, and mechanical degradation of PAM are slow and do not lead to acrylamide monomer. Low adsorption may be due to high solubility and smaller size (Guezennec et al., 2015). PAM is of three types, cationic, non-ionic, and anionic. Cationic absorbed most effectively while anionic is least absorbed, similar is their effectiveness for stabilisation since the negatively charged polymer is repelled by negatively charged clay particle, so polyvalent cation is used as 'cationic bridge' to counterbalance the negative charge of both clay and polymer (Seybold, 1994). Net charge, charge density, and molecular weight must be considered while selecting PAM along with soil properties. It is more effective in kaolinite than illite and significantly less in the case of quartz. Anionic PAM is more effective at lower pH (McLaughlin and Bartholomew, 2007), whereas Tekin et al. (2005) found an increase in adsorption with increasing pH.

## 3 Discussion and future scope of research

In recent years, the rapid development of infrastructures in metro cities compounded with the scarcity of useable land had compelled the engineers to improve weak soil properties to bear the load transferred by the infrastructure, e.g., buildings, bridges, roadways, railways, etc. The purpose of ground improvement techniques is to increase the bearing capacity of the soil, modify the sub-surface hydrology, and reduce the settlement to a considerable extent. Various techniques are available and evolving with time, including mechanical, biotechnical, and chemical improvement. Nowadays, various types of chemical stabilisation methods are available and widely practised, primarily for highways. Chemical methods are advantageous over mechanical soil stabilisation methods in many aspects like they do not produce noise and vibrations, unlike dynamic compaction. Though some degree of risk related to the environment is always there, it could be prevented by some extra carefulness. The inorganic waste material used for ground improvement possesses a potential threat of leaching heavy metals; there is a need to develop chemical compounds that could bind these heavy metal ions in solid-state or form a harmless complex. Similarly, for polymeric compounds sincere efforts to be made to address hazards of epoxy resins, PAM and PU. Since chemical stabilisation brings permanent changes in soil properties by altering its composition, they are more effective in expansive clays. Chemical stabilisation mostly uses industrial byproducts as a stabilising agent, thus also helpful for waste disposal, and it could reduce the overall cost of the project. Despite all these advantages, the soil being a heterogeneous material with varying mineral compositions, whereas most of the chemical stabilisation methods depend on chemical composition and mechanical properties of soil, special care must be taken in terms of both laboratory and field investigation before its application. Methods such as cement or lime mixing may be exempted due to a large amount of data available, but newly developed and less practised methods should be rigorously studied.

Biochemical methods are usually preferred for sandy soil since the movement of bacteria is restricted by the size of pores. Large pore space is suitable for travelling of bacteria, but as the pore space increase, particle-particle contact decreases, which is the site for cementation, and hence the strength decreases. An optimisation technique must be developed to balance the two opposing parameters to get maximum strength, structural arrangement, and the size of the particle should be considered before application of the method. For selecting the microbes, the bacteria which can sustain in high concentration ammonia and having no pathogenic effect should be taken. Calcite precipitating and low urease activity bacteria are the most preferred ones. The calcite precipitation rate should be such that neither it blocks the inlet nor takes too long to stabilise. The fact that bacteria can survive over a long time supports this method's utility, but it is difficult to predict their growth, spread, and concentration. Thus, some kind of testing or monitoring has to be developed, which can test/monitor the growth, spread, and concentration of microbes over time. A large number of tests should be conducted to produce sufficient data to predict the outcome of the application of different kinds of microbes, along with focus should be on the development of a sophisticated monitoring system to ensure the achievement of desirable uniform strength over an entire area. However, biochemical methods provide a better alternative than traditional mechanical ones since it does not produce any sound or vibrations during treatment and harmful chemical unlike the high amount of CO_2_ liberation in cement production. Also, this method works best at room temperature, and the strength keeps on increasing with time due to the development of the microbial cluster. Apart from subsurface stabilisation, it can effectively bind the surface soil resulting in a decline of soil erosion and helping in dust suppression. However, proper care must be needed to avoid excessive water infiltration, and inundation must not take place as it may wash out microbe and the occurrence of acid rain can dissolve precipitated Calcium. Thus, there is a need for developing a proper drainage network in the stabilised area. Effects of temperature, mineralogy of soil, ecology, moisture content, and climate need to be studied to increase the method's effectiveness.

Similarly, the use of various bio-enzymes for ground improvement is restricted by the type of soil. Although a particular bio-enzyme is suitable for a specific soil type, it should never be used alone. Instead, it should be used as a secondary stabiliser, e.g., with cement. Entirely relying on them would not be a good idea. Another factor that must be kept in mind before applying these techniques is the availability of time. Since the development and growth of a bacterial colony takes some time, these methods are not advisable in projects requiring immediate ground improvement. The focus must be on identification of various types of microbes which can sustain over a wide range of geological condition and can develop desired cementing property in the shortest time.

The electrochemical stabilisation method is best suitable for the removal of water, increasing bearing capacity, and consolidation of expansive clays, especially when removal of soil or ex-situ mixing is not feasible. It utilises the concept of movement of electrolytic water under the influence of an electric field. Copper-based electrodes can be used to reduce the loss of power; however, the use of EVD nowadays making it more economical. Simultaneous injection of chemicals such as Sodium silicate and Calcium Chloride at electrode brings long term hardening and workability of foundation. Polarity reversal may or may not work depending on setup, mechanism of stabilisation, and chemicals used. Changes in pH and unexpected electrochemical changes in the soil are some reasons for the hesitation of engineers to deploy this method which can be overcome with some detailed study and field testing. Precipitation of minerals, electrolysis of water, and accumulation of gases near electrode are some problems associated with the method. Still, they can be controlled with proper planning and execution, e.g., accumulation of gas can be control with EVD. While using this method, due attention must be given to voltage gradient and pore size. At a high voltage gradient, efficiency decreases, whereas, in large pores, electrolysis dominates. Thus, further research is needed to develop a high voltage efficient method, which will drastically increase this acceptance method. Another problem generally encountered while using this technique is the non-uniform settlements. In-depth research is required for achieving a uniform settlement and uniform increase in bearing capacity, not only near an electrode. Comparative study of various combinations of electrode positions should be conducted to get the optimal spacing for the required outcome. The effect of mineralogy, and which type of ions are more suitable in what type of soil with what mobility, should be studied to get a closer insight of the method, also what harmful effects are likely to appear according to mineralogy, and how they are going to affect the ecology and ecosystem should be studied with possible solutions. Some cheaper material is needed to be developed as electrodes so the cost of installation can be reduced. Also, this technique fails to give desire results in the absence of surface conductance which needs to be resolved.

Chemicals such as cement, lime, and fly ash are primitive methods of soil stabilisation. Much data is available, which could be helpful in the prediction of their effects. However, there is a need for the development of cement, which does not increase the brittleness of soil to a larger extent. Apart from these, cheap and more environmentally friendly (lesser emission of CO_2_ during production) cement will always be welcomed. Cation exchange, flocculation, pozzolanic reaction, and cementitious hydration are the supposed mechanism of stabilisation. Cation exchange and flocculation occur within a short time, reducing swelling immediately, while pozzolanic reaction and cementitious hydration which gradually hardens the soil, takes a sufficiently long time. The effect of the addition of a binder is not the same all the time. Sometimes both cohesion and friction angle increase, and sometimes only one parameter increase and other decrease but the overall strength of soil always increase. So, before the application, one must check the performance of the site. High pH is suitable for these methods as it dissolves silica, forming Silicate Hydrate with Calcium and Aluminium. Further research must develop a cement composition that could be effectively used in an acidic (at least mild acidic) environment. At higher concentration, cementation dominates, whereas at lower concentration flocculation, and cation exchange occurs. More detailed study and testing are required to add a few readily available, economical, and environmentally friendly materials such as rice husk ash, coconut shell ash, fly ash and blast furnace slag as a substitute for cement. In the case of fly ash, Class C fly ash is usually used due to the high amount of Calcium, whereas Class F fly ash can be used along with lime to augment the scarcity of Calcium. Sometimes due to the unavailability of a complete mixing setup, the mixture of soil and binder (FA, lime, or cement) are required to be store for some time, or they are transported from one place to another. This may cause a delay in compaction, which results in lesser MDD as cementation starts after mixing the binder with soil, and some effort of compaction goes to break the cementitious bond. To delay the cementation process, engineers are advised to use chemicals such as modified lignosulfonate which delays the onset of the cementation process on their addition. There is a need to establish a relationship between settling time and soil type (mineralogical composition) which will be helpful in the transportation of cement soil mix. If the water requirement could be reduced, it will be highly beneficial, especially in the dry climate. Leaching of heavy metals into groundwater in case of fly ash needs better addressing. The presence of humus and organic matter along with Sulphate attack and Carbonation, are some of the factors that severely affect the utility of cement and lime-based stabilisers. Thus, before application, the study of mineralogy and composition of ground must be carried out.

Like FA, CCR is also a waste product containing Ca(OH)_2_ as the major constituent and pozzolanic binders such as Ca and Al. Its application increases pH and dissolves silica, thus providing favourable conditions for the pozzolanic reaction. It reduces double layer thickness by cation exchange. Its use as a stabilisation agent solely is not advisable; instead, it may be effectively used to augment fly ash, mostly class F fly ash, as it contains Calcium which Class F FA generally lacks. Similarly, various other inorganic materials such as Sodium Silicate and Calcium Chloride, some salts, e.g., CaCl_2_, NaCl etc., can also supplement other stabilisation techniques. Theoretically, their application will improve the overall efficiency of various traditional stabilisation techniques, which needs a detailed field cum laboratory study before large-scale implementation.

The use of Polyurethane as a grouting material is still in a nascent stage; many scopes is there for improvement. Though it is expansive, it does not increase brittleness, provide good post-failure strength, reduce erodibility, and incorporates ductility while increasing bearing capacity. The stabilisation generally takes place via two mechanisms, it mixes with soil and covers soil particle, thus increases bond strength, and it swells, thus filling the soil voids and displace the water. Due to its ability to fill up the pore space, it can also lift the foundation. Due to its inherent viscosity, it is difficult to inject it in clays and fine-grained soil as it requires very high pressure. Dilution could be helpful as it results in better permeability, but with an increase in dilution, binding capacity decrease. So, obtaining a desired workability within the economic value, a trade-off must be obtained between dilution and desire strength. Longer time taken during polymerisation reaction is another crucial factor and efforts should be made to increase its reaction time without compromising its effectiveness. Continuous improvement in its strength by adding fillers and chain extenders is going on. A lot of efforts are needed to address problems due to water to make it worthwhile for wet soils. Due to its flexible nature, it may prove to be helpful to attenuate waves, so its effects when the ground is subjected to dynamic loading should be studied in detail.

Lignosulfonate is an environmentally friendly binder produced as waste from the wood and paper industry. Its application does not increase the pH and brittleness of soil and also does not alter the permeability of the soil. It helps decrease the double layer thickness, soil erosion and can also be used for dust suppression. Like bio enzyme, it is also not self-sufficient for high strength development thus must be used along with other stabilisers. Polyacrylamide is a water-soluble polymer. Although its monomer unit is highly toxic, the developed polymer is harmless. Since no polymerisation occurs to the full extent, some monomers can remain in a polymer binder, threatening the environment. Thus, the use of it is generally limited to areas far away from human settlements. It is mostly used to aggregate soil particles so the soil does not erode and remains permeable. Higher polymers are more effective in developing high permeability, thus reduces the risk of the development of high pore pressure. Another polymer that can be effectively used for stabilisation is epoxy resin. It is generally used with a hardener and is very stable, and increase the ductility of soil. The epoxy resins provide good post-failure strength and strain hardening; their high permeability makes them favourable for injection grouting.

LS, PU, and Biochemical methods should be explored for dust suppression, especially in opencast mines and ongoing construction sites. Methods based on biogas could be useful in preventing damages in case of ground vibration as biogas generating bacteria reduce the capacity of soil to hold water, and attenuation of the wave is exponentially fast as the amount of gas increase in voids resulting in lesser peak particle velocity (Wang et al., 2004). Similar effects from PU are also expected as it is more flexible and could attenuate waves. The development of thin permeable cement-based grout will revolutionise ground improvement since handling of extensive material will be replaced with an economical method, especially for fine clays. Similarly, the use of Sodium Silicate as a stabiliser can be fruitful since it is thin, economical, and harmless, but many times soil starts losing strength due to its solubility; if this issue can be addressed, it may be a suitable replacement for other grouts.

Detailed literature studies indicate the utility of using chemical stabilisers in improving the ground condition. One of the most critical aspects of chemical stabilisers is that they are primarily used in combinations within themselves or other stabilisation methods. However, few chemical techniques can be used alone, but the rest of them acts as augmentation to amplify, speed up, and increase the effectiveness of ground improvement measures. The use of waste materials such as fly ash and calcium carbide residue not only improves the strength of soil but also provides a safer way of waste disposal. Achieving a uniform composition and strength improvement is another challenge since the treated ground can have some weak spots. In some cases, an area with higher additive concentration due to miss application also poses a critical problem, e.g., in cement, where an excess amount of cement gives rise to high brittleness. So, proper care should be taken for uniform mixing, and it should not be beyond the optimum value. Table 1 summarises the effect of using various chemical stabilisers in improving the overall strength of the soil. It is difficult to propose a specific stabilisation method for a particular soil type, but Table 2 presents some favourable and unfavourable soil type for different types of stabilisation.

**Table 1:** Utility of various chemical stabilisation methods in improving ground properties.

Chemical Stabilisers	Change in UCS (kPa)		Change in C (kPa)		Change in Ø (°)		Note	Source	Curing time (days)	Quantity
TerraZyme	450	2200					Black cotton soil	Navale et al. (2019)	14	300ml/1.5m^2^
Bio enzyme	140	185	125	185			Mountain soil	Moravej et al. (2018)	7	400 ml/m^3^
Terrazyme	117	125					Illite	Khan et al. (2015)	28	defined by supplier
*Bacillus subtilis*	12.74	34.3	49	137.2	4.46	35.07	Sandy clay	Harianto et al. (2013)	28	9cc
*Rhizopus oligosporus*			0	24	27	25	Sand	Lim et al. (2020)		
*B. Megaterium*			23	61.7	44.2	51.6	Sandy silt	Min Le (2015)		
*B. Megaterium*	50	125	23.8	61.7			Tropical residue	Soon et al. (2013)		
Ureolytic bacteria	850	2067					Fine sand	Cheng and Ruwisch (2014)		
Electroosmosis			174	121	24	30.9	Ca ion	Abdullah and Al-Abadi (2010)		
Fly ash			152	195	20	35	Expansive clay	Hasan (2012)	21	10%
Fly ash	24.73	63.38					Clayey soil	Sharma et al. (2012)		20%
Fly ash	212	713						Bin-Shafique et al. (2010)		10%
Fly ash	425	1230					At OMC	Misra (1998)		20%
Fly ash			24.5	33.32	30°15′	34°12′		Prabakar et al. (2004)		35.50%
Fly ash			38.25	20.1	29	37		Rajak et al. (2019)		30%
Rice husk ash			60	37.5	17°50′	31	Alluvial soil	Rathan Raj et al. (2016)		30%
Coconut shell ash			24.41	19.5	13	17	Lateritic soil	Amu et al. (2011)		6%
Lime + Fly ash			10	24	35	41	Sandy soil	Consoli et al. (2001)		4%lime+25 % FA
Lime	171	288					Sodic soil	Umesha et al. (2009)		3%
Lime	312.04	1350					Compressive clay	Jha and Sivapullaiah (2015)		6%
Cement	400	4300						Asgari et al. (2015)	21	7%
Cement	1000	3500–5000						Akpokodje (1985)		12%
Calcium carbide residue	800	2100					Silty clay	Kampala and Horpibulsuk (2013)	7	6%
Calcium carbide residue	78.4	855.54					Expansive clay	Isah and Sharmilab (2015)		4%
Calcium carbide residue	300	1400–1800						Latifi et al. (2018)		
Calcium carbide residue	286	1900						Latifi and Meahen (2017)	90	9%
Silica fumes	48.98	198.65	37	120	7	11	Expansive soil	Singh et al. (2020)		40%–60%
CaCl_2_	49.5	311.8						Krishna and Ramesh (2012)		
Polyurethane	125	216						Saleh et al. (2020)		
Polyurethane	39.43	50.94	0.11	0.87	7.82	8.46	1:1 sand cyay mix	Liu et al. (2012)		5%
Polyurethane	0/difficult to make sample	131.87	4.32	36	30.12	34.56	Sand	Liu et al. (2019a)		30%
Polyurethane			75	250			Marine clay	Saleh et al. (2018)		5%
Polyurethane	66	145					Marine clay	Saleh et al. (2020)	3	6%
Lignosulfonate	120	130						Sezer et al. (2016)		2%
Lignosulfonate	338	496	235	266	10	15	Expansive clay	Noorzad and Ta'negonbadi (2018)		4%
Lignosulfonate	600	1400					Na LS, bauxite dust	Ding et al. (2018)		10%
Lignosulfonate	610	776					Ca LS, lateritic soil	Ravishankar et al. (2017)	28	2%
Epoxy resin	54.831	166.6						Kumar and Kumar (2015)		5%

**Table 2:** Applicability and non-applicability of various chemical stabilisers to different soil types.

Method	Applicability	Unsuitable Soil type
MICP	Medium size sandy soil	Soil with a too large or too small pore size
Bio-Enzyme	Highly soil specific	
Electro-Osmosis	Suitable for fine, Impermeable clays	Soils possessing high carbonate buffer and high cation exchange capacities
Cement and Lime		Organic soil with low pH
		Gypsiferous/Bassanitic soils
Fly Ash		Organic soil with low pH
CCR	Clays	
Silica Fumes	Expansive soils	
Sodium Silicate-based stabilisers	Sulphate containing soils which are otherwise problematic with traditional stabilisers such as lime, cement, and fly ash	
NaOH + CaCl_2_	Useful for in situ improvement	
PU	Repairing highway pavement	Care must be taken when using in wet soil
	medium size sand	Clayey soil
	Sealing cracks or for ground improvement where strata bear a large amount of water	
	When high brittleness may be dangerous	
LS	Minimise disturbance to ecology	
	Dispersive clay	
Epoxy resin	Dispersive clay	
	Reduction in permeability may be harmful	
PAM	Highway slopes	
	Erodible soil	
	Reduction in permeability may be harmful	

## 4 Conclusions

Ground improvement plays an important role in the construction of various structures such as large Skyscrapers, Dams, Petroleum storage facilities, and Highways. There are a lot of challenges for engineers in the field of ground improvement. There is a strong need to develop more reliable, environmentally friendly, and less time-consuming methods. Some old technologies are working flawlessly and some are under the developmental stage, but there is a need for more application-oriented methods which can be applied to different types of problematic soils. In this study, some of these challenges have been identified. This work is an attempt to review the development of ground improvement techniques over the period, mainly focusing on chemical stabilizers. Traditional and conventional stabilizers are discussed along with their applicability condition and potential hazards associated with them.

Among the fundamental problems requiring further research includes:

➢ The selection criterion of suitable technique for ground improvement, their long term serviceability, and effect of different ecological and environmental factors.
➢ The focus should be on developing practical, economical, sustainable, and environmentally safe chemical stabilization methods considering carbon emissions and overall costs including environmental costs.
➢ How can industrial waste materials be judiciously used in ground improvement, providing an economical alternative to traditional techniques, and facilitating their disposal?
➢ The harmful effects of using chemical stabilizers must be studied, and proper care and design must be carried out before the project's execution.
➢ There is an optimal concentration for every chemical 985 stabilizers for delivering the best performance. This has to be worked out before the execution of any project
➢ Although chemical stabilizers provide many advantages over traditional mechanical stabilization like reduction in noise, vibrations, and dust, their use, storage, handling, and application require specialized training
➢ It must be noted that the best utility of the chemical stabilizers is obtained when they are used in combination rather than relying on any particular stabilizer.
➢ The usefulness of these methods requires the development of techniques for long-term monitoring of strength improvement of the treated ground and tracking any adverse effect on the environment. Long-term monitoring is still missing in the field of ground stabilization.

The future research and field experience such as above will further help the sub-discipline of ground improvement to grow. Its development and importance is a critical component of successful geotechnical engineering and construction processes.

## Declarations

### Author contribution statement

All authors listed have significantly contributed to the development and the writing of this article.

### Funding statement

Rajesh Rai was supported by Northern Coalfields Limited, Singrauli, India.

Rajesh Rai is grateful to for providing financial support.

### Data availability statement

Data included in article/supplementary material/referenced in article.

### Declaration of interests statement

The authors declare no conflict of interest.

### Additional information

No additional information is available for this paper.
